# Re-innervation of neuromuscular junctions by a conductive polypyrrole/silk fibroin/GelMA hydrogel facilitated functional skeletal muscle regeneration following volumetric muscle loss

**DOI:** 10.1016/j.jot.2026.101128

**Published:** 2026-05-15

**Authors:** Dimulati Maimaiti, Ziying He, Jinuo Liu, Shihan Gao, Guanyu Yang, Ce Qi, Kai Meng, Fan He, Huijing Zhao, Xi Chen

**Affiliations:** a Department of Pathology, Third Affiliated Hospital of Soochow University, Soochow University, Changzhou, 213003, China; b School of Basic Medical Sciences, Suzhou Medical College, Soochow University, Suzhou, 215007, China; c Orthopaedic Institute, Suzhou Medical College, Soochow University, Suzhou, 215007, China; d National Engineering Laboratory for Modern Silk, College of Textile and Clothing Engineering, Soochow University, Suzhou, 215123, China; e Department of Orthopaedics, First Affiliated Hospital of Soochow University, Soochow University, Suzhou, 215006, China

**Keywords:** Skeletal muscle regeneration, Conductive hydrogel, Volumetric muscle loss, Angiogenesis, Re-innervation, Peripheral nerves

## Abstract

**Introduction:**

Volumetric muscle loss (VML) is a significant clinical challenge that severely compromises patients' motor function and often results in irreversible disability. While conventional hydrogels have been explored for VML repair, their inability to address peripheral nerve denervation has limited functional recovery.

**Objectives:**

The objective of this study was to develop a conductive double-crosslinking hydrogel (PPY@SF/GelMA) by integrating polypyrrole (PPY) into gelatin methacryloyl (GelMA) and silk fibroin (SF), aiming to simultaneously promote myotube formation and nerve re-innervation.

**Methods:**

The micro-architecture, compressive strength, rheological properties, swelling behavior, and conductivity of the PPY@SF/GelMA hydrogel were assessed. The influence of the conductive hydrogel on *in vitro* myogenic differentiation of C2C12 myoblast cells and angiogenic differentiation of endothelial cells was evaluated. The *in vivo* biodegradation and biocompatibility of the conductive hydrogel were assessed through subcutaneous implantation in the dorsal region of C57BL mice. The regenerative potential of the conductive hydrogel for skeletal muscle and peripheral nerve repair was investigated using a mouse tibialis anterior VML model.

**Results:**

Compared to pure GelMA or SF hydrogels, the PPY@SF/GelMA composite exhibited superior mechanical resilience, tunable swelling kinetics, exceptional biocompatibility, and enhanced electrical conductivity. *In vitro* experiments using C2C12 murine myoblasts demonstrated that the PPY@SF/GelMA hydrogel markedly upregulated myogenic differentiation markers (e.g., *Mhc*, *Myog*, and *MyoD*) and promoted the formation of multinucleated myotubes. Additionally, the conductive hydrogel exhibited pro-angiogenic potential by enhancing endothelial cell differentiation, as evidenced by new formation of endothelial tubes. *In vivo*, histopathological analysis showed no signs of toxicity from the implanted conductive hydrogel. PPY@SF/GelMA implantation facilitated the regeneration of aligned muscle fibers, reduced fibrotic collagen deposition, and accelerated neovascularization. Importantly, the conductive hydrogel successfully promoted peripheral nerve re-innervation by restoring neuromuscular junctions. RNA-seq analysis further revealed the involvement of the phosphoinositide 3-kinase (PI3K) signaling pathway in the newly regenerated muscle treated with PPY@SF/GelMA.

**Conclusions:**

Our findings demonstrate PPY@SF/GelMA as a promising therapeutic scaffold to facilitate both myogenic and neurogenic regeneration after VML injuries, offering a translatable strategy for complex musculoskeletal repair.

**The translational potential of this article:**

The failure of skeletal muscle regeneration following VML injuries is primarily attributed to the loss of peripheral nerve innervation. The present investigation has demonstrated that the conductive PPY@SF/GelMA hydrogel effectively promoted myofiber maturation and restored neuromuscular junctions, thereby promoting the re-innervation of peripheral nerves in newly regenerated skeletal muscle. Our objective is to translate this conductive hydrogel into a viable clinical strategy for patients requiring the repair of severe muscle damage.

## Graphical abstract

**Figure:**
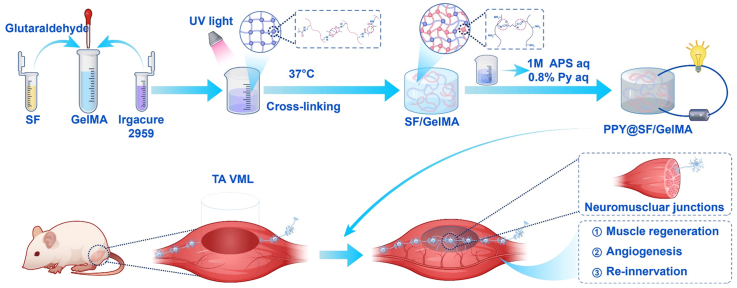

## 1 Introduction

Skeletal muscle, a highly structured tissue within the human body, constitutes over 40% of total body mass and is essential for facilitating voluntary movement. Under normal physiological conditions, skeletal muscle exhibits an intrinsic regenerative capacity, enabling it to repair minor injuries effectively [1]. Nevertheless, this innate self-repair mechanism is compromised by substantial muscle tissue loss due to traumatic events, such as accidents, warfare, or surgical resection. These injuries are characterized as volumetric muscle loss (VML), which affects more than 20% of an individual's muscle volume, resulting in significant scar tissue formation, impairment of locomotor function, and potentially permanent disability [2]. At present, the transplantation of autologous muscle flaps, which contain microvascular and peripheral nerve tissues, is the standard treatment for extensive VML injuries to promote skeletal muscle reconstruction. Nonetheless, recovery following VML remains constrained due to the scarcity of autologous muscle grafts and the morbidity associated with donor sites [3].

Recently, extensive attentions have been paid to hydrogels, as these biomaterials present significant potential for tissue regeneration and the restoration of normal muscle function. The favorable biophysical properties of hydrogels not only mimic the microstructure of the native extracellular matrix (ECM) but also create a suitable microenvironment for muscle progenitor cell survival, proliferation, and differentiation at the site of injury [4]. Natural biopolymers, such as collagen, gelatin, chitosan, and hyaluronan, have been engineered for targeted applications in skeletal muscle regeneration due to their favorable biocompatibility, adjustable structural properties, and adequate mechanical support. A prior study conducted in our laboratory demonstrated that gelatin methacryloyl (GelMA) hydrogel, when combined with an endogenous hormone melatonin, enhanced the formation of mature myofibers by augmenting the energy metabolism of myoblasts in a rat model of VML [5]. Furthermore, silk fibroin (SF), a natural protein extracted from silk cocoons, has recently been utilized in the fabrication of biomaterials and the repair of skeletal muscle [6]. Park et al. demonstrated that an increase in SF content within polyurethane correspondingly enhanced the expression of muscle marker genes in mouse C2C12 myoblast cells, suggesting a positive influence of SF on the differentiation of myoblast cells. Significantly, SF-based scaffolds have demonstrated the potential to facilitate the formation of new blood vessels lined with CD31-expressing endothelial cells, thereby supporting functional tissue repair in a rat model of onlay esophagoplasty [7]. Given the diverse physical properties of hydrogels, the combination of GelMA and SF with a dual cross-linking network presents itself as a promising biomaterial for skeletal muscle repair by providing enhanced mechanical support.

The functional reconstruction of extensively damaged skeletal muscle, with the capability for voluntary movement, necessitates the effective repair of peripheral nerve tissues [8], because the contractile response of skeletal muscle tissue is regulated by electrical signals transmitted via motor nerve action potentials [9]. Loss of innervation in skeletal muscle leads to a marked reduction in contractile capacity, primarily due to myofiber atrophy [10]. Transcriptomic analysis of vastus lateralis muscle biopsies from 575 participants in the large cohort Study of Muscle, Mobility, and Aging (SOMMA) has revealed that genes responsive to denervation are negatively correlated with muscle volume and performance. This finding underscores the critical role of innervation in determining muscle and mobility outcomes in aging individuals [11]. Although satellite cells maintain the potential for muscle regeneration following the loss of innervation, the concurrent occurrence of muscle injury and denervation leads to delayed maturation of newly formed myofibers. This delay is accompanied by alterations in the myofiber secretome, including changes in osteopontin and transforming growth factor-beta 1 (TGF-β1) levels [12]. Consequently, the timely restoration of neural innervation is essential for preserving skeletal muscle function. Passipieri et al. demonstrated that the implantation of a polycaprolactone (PCL) nerve conduit combined with adipose-derived stem cells not only effectively repaired surgery-induced injury to the rat common peroneal nerve but also successfully retrieved muscle contraction function by preventing long-term atrophy [13].

Given the critical role of innervation in skeletal muscle regeneration, numerous strategies have been employed, including electrical stimulation [14], stem cell transplantation [13], and three-dimensional (3D) printed constructs [15]. Recently, conductive hydrogels have attracted great attention for their potential to facilitate peripheral nerve regeneration by restoring the transmission of neural signals. Hydrogels integrated with conductive substances such as polyaniline (PANI) and carbon nanotubes (CNTs) can substantially enhance neural cell differentiation and nerve extension. Polypyrrole (PPY), a heterocyclic organic polymer, is noted for its excellent biocompatibility and high conductivity, making it suitable for applications in nerve regeneration, particularly in the form of biosensors and drug delivery systems [16]. A hydrogel composed of PPY-alginate copolymers has demonstrated the ability to enhance P19 neural cell differentiation by augmenting the induction of electrical impulses [17]. Notably, C2C12 myoblast cells cultured on a flexible PPY-deposited polydimethylsiloxane (PDMS) film showed a significant increase in myogenic gene expression and myotube formation under electrical stimulation [18]. To improve the degradation properties of PPY, Xuan et al. employed pyrrole-1-propionic acid to graft onto a hyaluronic acid (HA) hydrogel through a cystamine-containing disulfide bond. The HA-modified conductive hydrogel demonstrated a positive impact on Schwann cell myelination and peripheral nerve regeneration in a rat model of sciatic nerve crush injury [19]. Nonetheless, the therapeutic efficacy of a PPY-incorporated conductive hydrogel in repairing VML-injured skeletal muscle, as well as the underlying mechanisms, remains insufficiently understood.

**Scheme 1:**
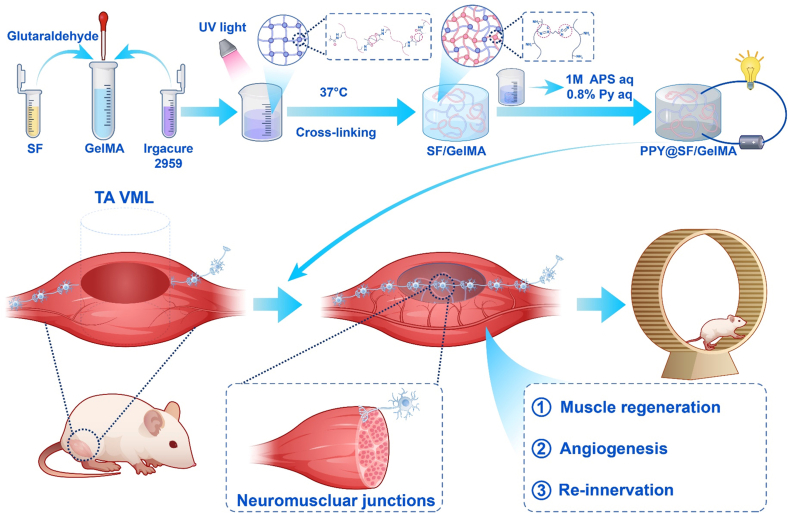
Schematic mechanism diagram of the PPY@SF/GelMA conductive hydrogel promoting vascularized skeletal muscle regeneration and peripheral nerve re-innervation.

The aim of this study was to develop a conductive double-crosslinking hydrogel by incorporating PPY with SF and GelMA (PPY@SF/GelMA) that can facilitate the re-innervation of peripheral nerves and restore motor function in VML-injured skeletal muscle. Initially, we synthesized a dual-network hydrogel through glutaraldehyde-mediated chemical crosslinking of SF protein and ultraviolet (UV)-initiated covalent crosslinking of GelMA. Subsequently, PPY was incorporated to enhance the hydrogel's conductive properties. Following this, the micro-architecture, compressive strength, rheological properties, swelling behavior, and conductivity of the PPY@SF/GelMA hydrogel were assessed. The impact of the conductive PPY@SF/GelMA hydrogel on *in vitro* myogenic differentiation of myoblast cells and angiogenic differentiation of endothelial cells was evaluated. Subsequently, the *in vivo* biodegradation and biocompatibility of the conductive hydrogel was evaluated through subcutaneous implantation in the dorsal region of C57BL mice. Finally, the regenerative potential of the conductive PPY@SF/GelMA hydrogel for skeletal muscle and peripheral nerve repair was comprehensively investigated using a mouse tibialis anterior (TA) VML model (Scheme 1).

## 2 Materials and methods

### 2.1 Materials

Silk used in this study was purchased from Minghe Sericulture Specialist Co-operative Society (Ningbo, China). Gelatin, methacrylic anhydride, glutaraldehyde (GA), ammonium persulfate (APS), and pyrrole were obtained from Macklin Biochemical Technology Co., Ltd (Shanghai, China). Anhydrous sodium carbonate (Na_2_CO_3_), lithium bromide (LiBr), sodium chloride (NaCl), potassium dihydrogen phosphate (KH_2_PO_4_, GR), and dicalcium phosphate (KCl) were purchased from Sinopharm Chemical Reagent Co., Ltd (Shanghai, China). Disodium hydrogenphosphate (Na_2_HPO_4_·12H_2_O) was purchased from Yuanye Bio-Technology Co., Ltd (Shanghai, China). Irgacure 2959 was purchased from IGM Resins Co., Ltd (Shanghai, China). Dialysis bags (MW cutoff: 3.5 kDa) was purchased from Suke Lean Instrument Co., Ltd (Suzhou, China). Anhydrous ethanol was purchased from Shanghai Lingfeng Chemical Reagent Co., Ltd (Shanghai, China).

### 2.2 Preparation of PPY@SF/GelMA

#### 2.2.1 Preparation of SF solution

Silk was introduced into a 0.2% sodium carbonate solution at a ratio of 1:200, subjected to boiling for 30 min with continuous stirring, and subsequently washed to remove silk sericin. The resulting SF solution was then dried in an oven at 60 °C for 4 h. A LiBr solution (9.3 M) was prepared and clarified through vacuum filtration. The SF solution was combined with the LiBr solution at a mass-to-volume ratio of 27:100, incubated at 60 °C for 4 h until completely dissolved, and then dialyzed for 48 h to remove impurities and metal ions. Finally, the SF solution was centrifuged for 10 min at 5500 rpm and store at 4 °C.

#### 2.2.2 Synthesis of GelMA

Gelatin was dissolved in 100 mL of phosphate-buffered saline (PBS, pH = 7.4) for 1 h at 60 °C. Subsequently, 8 mL of methacrylic anhydride was added and the mixture was stirred for 2 h at 50 °C. To terminate the reaction, 200 mL of PBS was added, and the solution was dialyzed for 5 d to eliminate unreacted methacrylic anhydride and methacrylic acid byproducts. The dialysate was then subjected to centrifugation at 3000 rpm for 10 min and subsequently lyophilized for 3 d. The resulting GelMA was dissolved in a 1.5% (w/v) Irgacure 2959 solution at a concentration of 100 mg/mL to yield a high-viscosity GelMA solution.

#### 2.2.3 Fabrication of PPY@SF/GelMA hydrogel

To synthesize the SF hydrogel, a 10% (w/v) SF solution was mixed with a cross-linking GA solution (10:1), followed by cross-linking at 37 °C. For the GelMA hydrogel, a 1.5% (w/v) GelMA solution was subjected to UV irradiation for 15 min. For the SF/GelMA hydrogel, a homogeneous mixture of 1.5% (w/v) GelMA solution and 10% (w/v) SF solution was prepared at a volume ratio of 1:1, and the resulting mixture was cross-linked under UV light for 15 min at 37 °C. The PPY@SF/GelMA hydrogel was synthesized through a sequential process involving the impregnation of the dual network hydrogel in an ammonium persulfate solution for 90 min. Subsequently, the hydrogel was immersed in a 0.8% pyrrole solution for 30 min. Following ultrasonic cleaning, the final PPY@SF/GelMA hydrogel was obtained by drying for 30 min at room temperature.

### 2.3 Characterization of hydrogels

#### 2.3.1 Scanning electron microscope (SEM)

The microstructure of hydrogels was observed by SEM (Hitachi, TM3030, Tokyo, Japan). Briefly, hydrogel samples were lyophilized after being frozen at −80 °C for 4 h, and then frozen with liquid nitrogen. Subsequently, gold was sputtered onto the cross sections of the constructs for 2 min. The cross-sections were observed under SEM at a 5 kV with 2000 magnification.

#### 2.3.2 Fourier transform infrared spectroscopy (FTIR)

The lyophilized hydrogels were placed in a mortar and pestle, ground into fine particles, and filled into an all-reflective platform. The FTIR spectra were recorded in the range of 500 to 4000 cm^−1^ at a scan rate of 80 spectra/sec using a spectrometer (Bruker, VERTEX 70, Saarbrucken, Germany).

#### 2.3.3 Mechanical property

The compressive characteristics of the hydrogels were evaluated utilizing a universal material testing machine (Instron 5967, Boston, MA, USA), operating at a compression rate of 2 mm/min until the point of fracture. The maximum compressive strength and maximum compressive strain were determined from the resulting compressive stress-strain curves. The compressive modulus was derived from the slope of the stress-strain curve within the 0-10% strain interval.

#### 2.3.4 Rheological properties

The rheological properties of the hydrogels were evaluated using an AR2000 DHR-2 rheometer (AR Instruments, Austin, TX, USA), employing a 20 mm steel parallel plate geometry at a temperature of 37 °C. The storage modulus (G′) and loss modulus (G″) were determined for hydrogel samples with dimensions of 2 mm in diameter and 1 mm in thickness. The shear moduli of individual samples were assessed at a frequency of 10 rad/s, with a strain range of 0.1-10% during the amplitude-sweep test. In the frequency-sweep test, the shear moduli of the samples were measured across a frequency range of 0.01-10 Hz at a constant strain of 1% [20].

#### 2.3.5 Swelling property of the hydrogels

The hydrogel specimens, each with a diameter of 14 mm and a thickness of 12 mm, were subjected to overnight freezing followed by lyophilization for 2 d. Initially, the mass of the freeze-dried hydrogels was recorded as M_1_. Subsequently, the samples were immersed in PBS at 37 °C and agitated at 100 rpm on a horizontal shaker to achieve swelling equilibrium. Upon reaching equilibrium, the mass of the swollen hydrogels, denoted as M_2_, was determined after carefully blotting away any excess surface water using filter paper. The swelling ratio of hydrogel was calculated by Equation (1):

(1) $$\text{Swelling}\;\text{ratio}\;\left(\%\right)=\left(M_{2}\;–\;M_{1}\;\right)\;/\;M_{1}\times 100\%$$

#### 2.3.6 Degradation rate analysis

The freeze-dried hydrogel samples were weighed (W_0_) and placed in PBS (pH = 7.4) with a solid-liquid ratio of 1:50, and the degradation system was placed in a 37 °C constant temperature water bath oscillator at 100 rpm. At the time points of 4, 8, 12, and 16 w, the hydrogels were rinsed with deionized water and then freeze-dried for 3 d. The weights of dried samples were recorded (W_d_) at each time point. The degradation ratio was calculated by Equation (2)

(2) $$\text{Degradation}\;\text{ratio}\;\left(\%\right)=W_{d}\;–\;W_{0}\;/\;W_{0}\times 100\%$$

#### 2.3.7 Conductivity property analysis

The SF/GelMA hydrogel and PPY@SF/GelMA hydrogel specimens were positioned between two copper electrode sheets, maintaining a clamping distance of 1 cm. Upon establishing an electrical circuit, the resistance of the hydrogels was measured using a multimeter (Owon B35T+, Zhangzhou, China), and their conductivity was subsequently calculated in accordance with Equation (3). To further validate the conductivity of the PPY@SF/GelMA hydrogel, it was integrated into a closed circuit with an LED bulb, which illuminated as expected.

(3) Conductivity=L/R·S

Where σ is the Conductivity (S/m), L is the distance between the two electrodes (m), R is the resistance (Ω), and S is the cross-sectional area of the hydrogel (m2).

#### 2.3.8 Elemental analysis

The nitrogen content within the conductive hydrogel was quantified using an elemental analyzer (Elementar Unicube, Frankfurt, Germany). The freeze-dried sample was subjected to complete decomposition in a high-temperature combustion tube, allowing for the detection of the nitrogen signal. The total nitrogen content was calculated using a calibration curve, and the mass fraction was determined according to the following formula.

(4) $$\text{PPy}\;\text{loading}\;\left(\text{wt}\%\right)=\frac{N_{\text{PPY}@\text{SF}/\text{GelMA}}‐N_{\text{SF}/\text{GelMA}}}{21.5\%}\times 100\%$$

#### 2.3.9 X-ray photoelectron spectroscopy (XPS)

An XPS analysis was conducted utilizing an X-ray photoelectron spectrometer (Thermo Scientific K-Alpha, Waltham, MA, USA). Dried hydrogel samples were carefully affixed to conductive tape, and measurements were performed using Al-Kα radiation (energy of 1486.6 eV) as the excitation source, under operating conditions of 12 kV and 10 mA. The resulting spectra underwent deconvolution fitting for analytical purposes.

### 2.4 In vitro experiments

#### 2.4.1 Cell culture and treatment

The murine C2C12 myoblasts were obtained from the Cell Bank of the Type Culture Collection of the Chinese Academy of Sciences (Shanghai Institute of Cell Biology, Shanghai, China). The cells were cultivated using high-glucose Dulbecco's modified Eagle's medium (DMEM) supplemented with 10% fetal bovine serum (FBS), 100 U/mL penicillin, and 100 μg/mL streptomycin (all from Thermo Fisher Scientific, Waltham, MA, USA). Upon reaching 90% confluence, the cells were switched to a myogenic differentiation medium containing high-glucose DMEM supplemented with 2% horse serum (Thermo Fisher Scientific). The medium was replaced every other day until fully differentiated myotubes was observed. To inhibit phosphoinositide 3-kinase (PI3K) activity, 10 μM LY294002 (GLPBIO, Montclair, CA, USA) was introduced into the culture medium, while cells in the control group received an equivalent volume of the vehicle, dimethyl sulfoxide (DMSO).

#### 2.4.2 Live/dead cell staining assay

To prepare the hydrogel leachate for all *in vitro* biological assessments, sterilized hydrogel samples were immersed in high-glucose DMEM at a concentration of 0.2 g/mL. The mixtures were incubated at 37 °C under continuous gentle agitation for 24 h to facilitate the release of soluble components and nanofragments. Subsequently, the leachate was collected by centrifugation at 12,000×*g* for 10 min, and the resulting supernatant was filtered through a 0.22 μm sterile membrane to remove debris and ensure sterility. The filtered extract was stored at 4 °C and used for subsequent cell culture assays within 24 h. The cell viability was determined using a live/dead cell staining kit (Beyotime, Haimen, China). C2C12 cells were seeded at a density of 5000 cells/cm^2^ in a 24-well plate and treated with the leachate of different hydrogels, while treatment with PBS served as the control group. At 1, 3, and 5 days post-treatment, the cells were stained with the kit for 30 min at 37 °C. The stained cells were observed and imaged using a Zeiss Axiovert 40CFL fluorescence microscope (Zeiss, Oberkochen, Germany).

#### 2.4.3 Cell proliferation assay

The cell proliferation was quantified using the Cell Counting Kit-8 (CCK-8) assay (Beyotime). The C2C12 cells were seeded at a density of 5000 cells/cm^2^ in a 96-well plate and incubated with the hydrogel leachate. At predetermined intervals of 1, 3 and 5 d, the cells were incubated in CCK-8 solution in the dark for 1 h at 37 °C. The absorbance at 450 nm was determined using a PowerWave XS spectrophotometer (BioTek, Winooski, VT, USA).

#### 2.4.4 Scratch wound assay

The C2C12 cells were seeded at a density of 5000 cells/cm^2^ in a 6-well plate and incubated with the leachate of different hydrogels. A scratch wound assay was conducted by creating a straight and gentle scraping using a sterile pipette tip. Following three times washes in PBS, the cells were incubated in the culture medium supplemented with the hydrogel leachate. The wound images were captured after 12 and 24 h using an optical microscope.

#### 2.4.5 Quantitative reverse transcription-polymerase chain reaction (RT-qPCR)

Total RNA was extracted using the FastPure® Cell/Tissue Total RNA Isolation Kit V2 (Vazyme Biotech Co., Ltd., Nanjing, China). The complementary DNA (cDNA) was prepared using the PrimeScript RT Reagent Kit (Takara, Tokyo, Japan) as previously described [21]. Quantitative PCR was conducted using the iTap™ Universal SYBR® Green Supermix kit (Bio-Rad, Hercules, CA, USA) on a CFX96™ Real-Time PCR System (Bio-Rad). The expression level of the target gene was normalized to that of the housekeeping gene glyceraldehyde-3-phosphate dehydrogenase (*Gapdh*) using the 2^−ΔΔCT^ method. The primer sequences are listed in Suppl. Table 1.

#### 2.4.6 Immunofluorescence staining

The immunofluorescence staining process entailed the initial blocking of tissue sections with 5% bovine serum albumin (BSA, Beyotime) for 60 min, followed by an overnight incubation at 4 °C with primary antibodies (Suppl. Table 2). Subsequently, the sections were washed three times with PBS and exposed to a secondary antibody for 60 min at room temperature in the absence of light. The nuclei were counterstained with 4′,6-diamidino-2-phenylindole (DAPI, Thermo Fisher Scientific). Three randomly selected fields of view were captured using a Zeiss Axiocam microscope. The proportion of positive areas was determined by calculating the ratio of the positive area using the ImageJ software (National Institutes of Health, Bethesda, MD, USA).

#### 2.4.7 Western blot

The total protein was extracted using a radioimmunoprecipitation assay (RIPA, Solarbio Science & Technology Co.,Ltd., Beijing, China) buffer containing protease inhibitors (Beyotime). The protein concentration was determined by using a BCA Protein Assay Kit (Beyotime). Equal quantities of protein samples were subjected to 10% sodium dodecyl sulfate-polyacrylamide gel electrophoresis (SDS-PAGE) and subsequently transferred to nitrocellulose membranes (Beyotime). Following the blocking step, the membranes were incubated with primary antibodies (Suppl. Table 3) overnight at 4 °C. On the subsequent day, the membranes were incubated with a horseradish peroxidase (HRP)-labeled secondary antibody (1:10,000, Beyotime) for 1 h at room temperature. The resulting bands were visualized using SuperSignal West Pico Substrate (Thermo Fisher Scientific) and the ChemiDoc Touch Imaging System (Bio-Rad). The intensity of the bands was subsequently analyzed using the ImageJ software.

### 2.5 In vivo experiments

#### 2.5.1 Subcutaneous degradation and *in vivo* biosafety evaluation

All animal experiments were approved by the Ethics Committee of Soochow University (SUDA20240927A05), and all operations were performed according to the National Institutes of Health guidelines. Twenty-eight C57BL mice (male, 2-month age, and weighting 20-25 g) were obtained from the Animal Center of Soochow University (Suzhou, China). Mice were anesthetized with 3% sodium pentobarbital (1.5 mL/kg body weight; Shanghai Merck Co., Ltd., China). The dorsal skin of the mice was shaved, and an incision was made at a consistent site. Subsequently, four groups of hydrogels (GelMA, SF, SF/GelMA, and PPY@SF/GelMA) were implanted subcutaneously (n = 4), and the incision was closed using 4-0 silk sutures [22].

For the *in vivo* degradation test, the initial weight of the hydrogels was recorded prior to subcutaneous implantation. The degradation profile was determined by measuring the residual weight of the hydrogels at 7 and 14 days post-operation. Gravimetric analysis was performed by comparing the residual weight to the initial values.

For the *in vivo* biosafety evaluation, mice were sacrificed two weeks post-implantation, and major organs, including the heart, liver, spleen, lungs, and kidneys, were collected. Hematoxylin and eosin (H&E) staining was conducted to assess tissue morphology. Organ samples from the untreated group served as a negative control for comparison [23]. To assess the impact of the conductive PPY@SF/GelMA hydrogel on hepatic function, blood samples were obtained from the ocular region of mice and transferred into centrifuge tubes. These samples were incubated at ambient temperature for 2 h to facilitate stratification. Subsequently, the samples underwent centrifugation at 1000 g for 10 min at 4 °C. The resultant supernatant was carefully transferred to a new centrifuge tube for further analysis. The activity of alanine aminotransferase (ALT) and aspartate aminotransferase (AST), and the concentration of blood urea nitrogen (BUN) were quantified using commercially available kits in accordance with the manufacturer's protocol (all assay kits were purchased from Nanjing Jiancheng Bioengineering Institute, Nanjing, China).

#### 2.5.2 Establishment of a mouse TA muscle VML model

A total of thirty adult male C57BL/6 mice (aged 2 months and weighing 20-25 g) were obtained from the Animal Center of Soochow University to evaluate the regenerative efficacy of the hydrogels in a VML model. The animal cohort was strictly allocated to satisfy the distinct tissue processing requirements of paired functional-histological analyses versus transcriptomic profiling. For functional and morphological evaluations, twenty-four subjects were randomly assigned to six experimental groups (Sham, VML, GelMA, SF, SF/GelMA, and PPY@SF/GelMA; n = 4 per group). At the four-week endpoint, these subjects underwent *in vivo* functional assessments followed immediately by tissue harvest for paired histological analyses.

After being anesthetized with 3% sodium pentobarbital, the mice were subjected to a VML injury by creating a surgical defect measuring 3 × 3 × 3 mm in the center of the TA muscle using a puncher. Approximately 50 μL of the hydrogel precursor was injected directly into the defect area. The injection volume was chosen to slightly overfill the cavity in order to compensate for the modest volumetric contraction that occurs during UV-initiated and glutaraldehyde-mediated cross-linking, and to ensure complete interfacial contact with the marginally retracted muscle edges. Immediately after injection, gentle tamponade was applied with sterile gauze to remove any excess fluid expressed from the defect and to facilitate close apposition between the hydrogel and the host tissue, without compromising the retention of the cross-linking hydrogel within the defect cavity. Subsequently, the skin was sutured and the mice received intramuscular penicillin (40,000 Units/mouse) for three consecutive days. All animals were housed under constant humidity (50–60%), temperature (22–24 °C), and a 12-h light/dark cycle (6 am to 6 pm).

#### 2.5.3 Muscle function analysis

The assessment of skeletal muscle contractile function and run distance was conducted over a 4-week period following VML surgery, utilizing the Dynamic Muscle Data Acquisition and Analysis System (Zhongshidichuang Science and Technology Development Co., Ltd., Beijing, China). For the purpose of evaluating contractile function, each animal's foot was firmly secured to a footplate connected to a dual-mode muscle lever system, with both the knee and ankle positioned at right angles. The placement of the needle electrodes was made just beneath the skin, ensuring that they were not inserted too deeply into the muscle and preventing the activation of the antagonist compartment. The muscles were stimulated at a frequency of 100 Hz at an intensity of 5 V. Throughout the course of the measurements, the body temperature was maintained at a constant 37 °C. To measure the maximum run distance, each mouse was individually placed in a cage equipped with a running wheel (setting to 20 m per min), allowing the mice to run continuously. Any contact made by the mice with the electrodes was recorded until the point of exhaustion, which was defined as the mouse making cumulative contact with the electric shock stimulator for 5 s.

Four weeks following implantation, the hind paws of the mice were coated with dye, and the animals were permitted to traverse a channel lined with white paper to document their footprints. To evaluate the recovery of muscular function, the grip strength of the hindlimbs was measured using a specialized grip strength meter designed for mice, which featured a metal grid or bar as the grasping target (Bioseb, Shenzhen, China). The forelimbs of each mouse were gently restrained using a gauze wrap. Subsequently, the mice were held by the base of their tails and allowed to grasp the target with their hindlimbs. The mice were then pulled backward horizontally at a consistent velocity until their grip was released. The apparatus automatically recorded the peak grip force.

#### 2.5.4 Histological analysis

At four weeks post-surgery, TA muscle samples were harvested from the VML defect site and subsequently preserved by freezing in melting isopentane (Sigma–Aldrich) at −80 °C. Subsequently, the samples were sectioned at a thickness of 10 μm using a cryostat maintained at −25 °C (CM 3050 S, Leica Biosystem; Wetzlar, Germany). For H&E staining, the sections were incubated in a solution of hematoxylin for 3 min, followed by an 1-min incubation in eosin solution (all reagents were purchased from Jiancheng). For Masson's trichrome staining, the sections were incubated in a solution comprising hematoxylin and lichun red acid (Solarbio) for 5 min. Digital images were captured using a bright-field microscope (Zeiss).

#### 2.5.5 Immunofluorescence staining

The tissue slides were initially subjected to a blocking step with 5% BSA for 60 min. Subsequently, the slides were incubated at 4 °C overnight using different primary antibodies. After incubation, the sections were washed with PBS and exposed to fluorophore-conjugated secondary antibodies for 60 min at room temperature in a light-protected environment. The nuclei were counterstained with DAPI to facilitate visualization. Randomly selected fields of view were captured using a Zeiss Axiocam microscope. The proportion of positive areas was determined by calculating the ratio of the positive area to the total injured area using Image J software.

#### 2.5.6 RNA sequencing analysis of newly regenerated TA muscles

For RNA sequencing analysis, a separate cohort of six mice (n = 3 for the VML group and n = 3 for the PPY@SF/GelMA group) was used. At 4 weeks post-surgery, the newly regenerated TA muscle tissues were harvested and rapidly frozen in liquid nitrogen to preserve RNA integrity. Total RNA was isolated from the muscle tissues using the TRIzol reagent, and RNA quality was evaluated using an Agilent 2100 Bioanalyzer (Agilent Technologies, Santa Clara, CA, USA). Subsequent transcriptome sequencing and data analysis were performed by OE Biotech Co., Ltd (Shanghai, China). In accordance with the manufacturer's instructions, sequencing libraries were constructed utilizing the VAHTS Universal V10 RNA-seq Library Prep Kit (Premixed Version). Next-generation sequencing (NGS) was conducted on a NovaSeq 6000 platform (Illumina, San Diego, CA, USA), and gene expression analysis was performed using the HTSeq-count software. Differentially expressed genes (DEGs) were identified based on a *P*-value of less than 0.05 and a fold change greater than 2 or less than 0.5. Subsequently, Gene Ontology (GO), Kyoto Encyclopedia of Genes and Genomes (KEGG), and Gene Set Enrichment Analysis (GSEA) analyses were performed on the DEGs using a hypergeometric distribution algorithm to identify significantly enriched functional terms and pathways.

### 2.6 Statistical analysis

Statistical analyses were performed using GraphPad Prism 9.2 software (GraphPad Software Inc., San Diego, CA, USA). Multiple group comparisons were conducted using One-way Analysis of Variance (ANOVA) followed by Tukey's post hoc test, while two-group comparisons were performed using Student's *t*-test. *P* values < 0.05 (∗) or < 0.01 (∗∗) were considered as statistically significant.

## 3 Results

### 3.1 Characterization of the double network conductive hydrogel

**Fig. 1:**
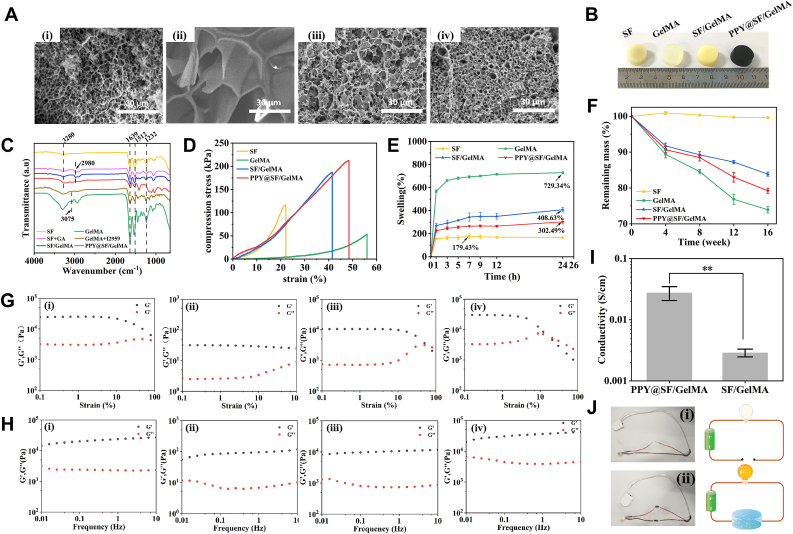
Characterization of the conductive PPY@SF/GelMA hydrogel (A) SEM image of ⅰ) SF, ⅱ) GelMA, ⅲ) SF/GelMA, and ⅳ) PPY@SF/GelMA hydrogels. Scale bar = 30 μm. (B) Gross morphology of the SF, GelMA, SF/GelMA, and PPY@SF/GelMA hydrogels (C) FTIR spectra of the four hydrogels (D) Compression stress-strain of the four hydrogels (n = 3) (E) Swelling ratio of the four hydrogels (n = 3) (F) Degradation rate of the four hydrogels (G-H) The rheological properties of ⅰ) GelMA, ⅱ) SF, ⅲ) SF/GelMA, and ⅳ) PPY@SF/GelMA hydrogels (I) The conductivity of the PPY@SF/GelMA and SF/GelMA hydrogels scaffolds (n = 3) (J) Schematic diagram of the circuit with LEDs when the PPY@SF/GelMA hydrogel was i) disconnected, ii) connected. Statistically significant differences are indicated by ∗ where *P*< 0.05 or ∗∗ <0.01 between the indicated groups.

To optimize the loading concentration of PPy, three conductive hydrogels were synthesized and subsequently incubated in pyrrole solutions at concentrations of 0.4%, 0.8%, and 1.2% for 30, 60, and 90 min, respectively. Conductivity assessments revealed that the hydrogels incubated in 0.8% and 1.2% pyrrole solutions demonstrated comparable conductivity levels, both of which were significantly higher than those observed in the 0.4% group (Suppl. Fig. 1A, Suppl. Table 4). A cell viability assay showed no statistically significant differences among the three groups (Suppl. Fig. 1B). XPS analysis identified the presence of sulfate ions (SO_4_^2−^) at binding energies of 168.7/169.9 eV and doped nitrogen at 401.2 eV within the PPY@SF/GelMA hydrogel, thereby confirming the establishment of a conductive PPy network (Suppl. Fig. 2). Elemental analysis and XPS characterization confirmed that the resultant PPy@SF/GelMA hydrogel comprised 7.74% (wt) PPy. SEM results showed that the four groups of hydrogels had a dense porous structure, which can support cell adhesion and migration as well as the nutrient exchange inside the hydrogels. Compared with pure GelMA hydrogel, the other three hydrogels possessed increased density and smaller pore sizes due to the cross-linking of SF and GelMA (Fig. 1A). The pore size was 4.38 ± 0.66 μm in the SF hydrogel, 5.65 ± 0.78 μm in the SF/GelMA hydrogel, and 3.15 ± 0.48 μm in the PPY@SF/GelMA hydrogel, compared with a big pore size in the range of 30 - 80 μm in GelMA hydrogel. With the supplement of PPY, the color of the PPY@SF/GelMA hydrogel changed to black (Fig. 1B). The FTIR spectra verified that bands at around 1637 cm^−1^ (amide I, C=O stretching vibration), 1512 cm^−1^ (amide II, N-H bending vibration and C-N stretching vibration), 1232 cm^−1^ (amide III), and 3280 cm^−1^ (-COOH) correspond to the characteristic peaks for silk fibroin. Meanwhile, bands at 2980 cm^−1^ (C=N) of the SF/GelMA hydrogel can be assigned to amino groups on the molecular chain of silk fibroin formed covalent cross-links with glutaraldehyde, while bands at 3075 cm^−1^ (N-H stretching vibration) and 1637 cm^−1^ (amide I band) confirmed that gelatin was chemically modified to form a gel. The characteristic peaks of SF proteins were not shifted and retained the β-sheet in amide I. Furthermore, the band at 1413 cm^−1^ in the PPY@SF/GelMA hydrogel can be ascribed to polymerized polypyrrole (Fig. 1C).

As shown in Fig. 1D, compression tests indicated that the mechanical properties of the SF/GelMA hydrogel as well as the PPY@SF/GelMA hydrogel were significantly improved by cross-linking SF and GelMA. The compressive modulus of the GelMA hydrogel was 3.20 ± 0.02 kPa, while the SF hydrogel reached 8.48 ± 0.03 kPa (Suppl. Table 5). However, the SF hydrogel was hard and brittle with less elastic deformation due to the internal β-sheet that restricted the movement of SF molecular chains. SF/GelMA hydrogels, combining the advantages of SF and GelMA with complementary properties, showed a moderate compressive modulus of up to 6.11 ± 0.02 kPa, with better compressive properties and elasticity. Furthermore, the mechanical properties of the PPY@SF/GelMA hydrogel were improved, with a compressive modulus of 5.61 ± 0.02 kPa and a maximum compressive strength of 212.47 ± 8.60 kPa, which was similar with the natural muscle matrix [24], holding the potential for skeletal muscle repair.

The swelling property is an important mechanical property of hydrogels (Fig. 1E). The GelMA gel showed the largest swelling ratio of 7.2-fold due to the large pore size and number of hydrophilic groups on the molecular chain. The maximum swelling rates of the SF, SF/GelMA, and PPY@SF/GelMA hydrogels were 179.4%, 408.6%, and 302.4%, respectively. With the addition of PPY, the pore size of the three-dimensional mesh structure inside the hydrogel became smaller, resulting in the reduction in the water absorption capacity. The reduced swelling rate and the improved stability indicated that the PPY@SF/GelMA hydrogel was capable to absorb and exchange nutrients required for cell culture. Furthermore, the *in vitro* degradation rate of the four different hydrogels within 16 weeks was shown in Fig. 1F. The degradation rate of the SF hydrogel was the slowest that was only 0.4 ± 0.1%, compared with the fastest degradation rate of the GelMA hydrogel (26.1 ± 0.7%), mainly due to the large amount of silk Ⅱ structure within the SF hydrogel. The degradation rate of PPY@SF/GelMA hydrogel was 17.4 ± 0.8%, slightly higher than that of the SF/GelMA hydrogel (16.1 ± 0.5%).

We next evaluated the rheological behavior of the four different hydrogels in the amplitude-sweep mode (Fig. 1G). The storage modulus (G′) of the GelMA hydrogel remained higher than the loss modulus (G″), indicating that the hydrogels exhibited a solid phase with the ability to maintain its morphology. It was observed that the modulus and strength of the PPY@SF/GelMA hydrogel were significantly increased. PPY can act as physical cross-linking points to strengthen the network structure of the conductive hydrogel through hydrogen bonding and hydrophobic interactions, thereby enhancing the storage modulus and mechanical strength [25]. The stability of hydrogels was further examined in the frequency-sweep mode (Fig. 1H). The storage modulus remained higher than loss modulus in a frequency range of 0.01-10 Hz, and the addition of PPY to the hydrogel effectively increased the crosslinking density and strength of PPY@SF/GelMA hydrogel. Furthermore, we examined the conductivity of the SF/GelMA and PPY@SF/GelMA hydrogels (Fig. 1I). Compared with the low conductive of SF/GelMA hydrogel (0.29 × 10^−2^ S cm^−1^), the addition of PPY significantly improved the conductivity of the PPY@SF/GelMA hydrogel (2.77 × 10^−2^ S cm^−1^). The successful lighting of an LED bulb verified the high conductivity of the PPY@SF/GelMA hydrogel (Fig. 1J).

### 3.2 Biocompatibility of the conductive double cross-linking PPY@SF/GelMA hydrogel

**Fig. 2:**
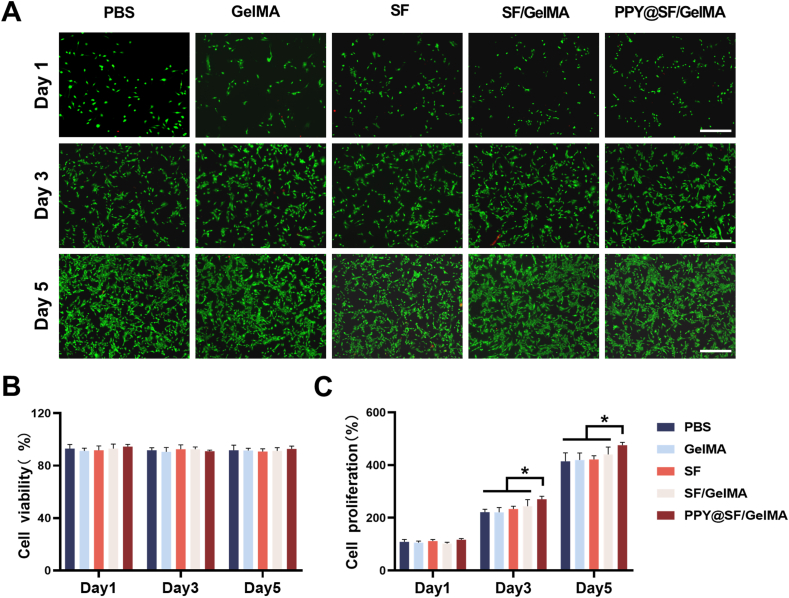
Biocompatibility of the hydrogels (A&B) C2C12 cells were treated with the extracted leachate of hydrogels, while the cells treated with PBS served as the CTRL group. Cell viability was evaluated using a Live/Dead staining assay (n = 3). Scale bar = 400 μm (C) The cell proliferation was quantified using the CCK-8 assay (n = 4). Statistically significant differences are indicated by ∗ where *P*< 0.05 or ∗∗ <0.01 between the indicated groups.

To investigate their biocompatibility, C2C12 cells were treated with the leachate extracted from GelMA, SF, SF/GelMA, and PPY@SF/GelMA hydrogels. Live/dead cell staining revealed a significant number of viable cells with minimal presence of dead cells (Fig. 2A and B). The CCK-8 assays demonstrated that the treatment with the PPY@SF/GelMA hydrogel significantly improved the proliferation of C2C12 cells by 16.6% at day 3 and 9.36% at day 5, respectively, compared with the PBS group (Fig. 2C). These results suggested that the four groups of hydrogels had favorable biocompatibility.

### 3.3 The PPY@SF/GelMA hydrogel improved the myogenic differentiation of C2C12 myoblasts

**Fig. 3:**
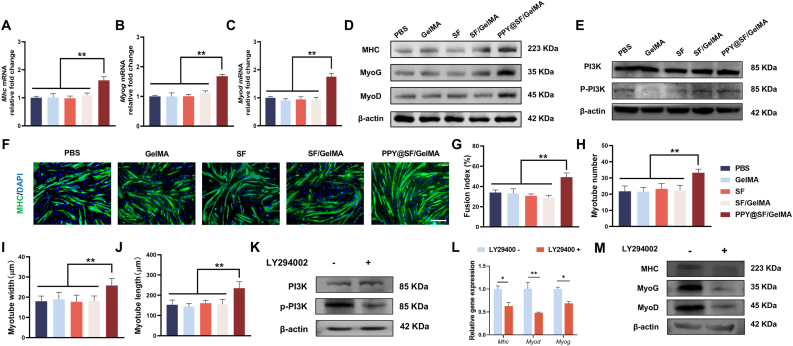
Effect of PPY@SF/GelMA on the myogenic differentiation of C2C12 cells (A-C) Quantitative real-time PCR analysis of the myogenic gene expression of myogenic markers, including (A) *Myod* (B) *Myog, and* (C) *Mhc*, was determined by quantitative RT-PCR (n = 4) (D) The protein levels of MHC, MyoG and MyoD were examined by Western blot (n = 3) (E) The effect of the conductive PPY@SF/GelMA hydrogel on the phosphorylation of PI3K (n = 3) (F) Immunofluorescence staining of MHC was conducted to evaluate myotube formation. Scale bar = 200 μm (G-I) Quantification of the (G) fusion index (H) numbers (I) width and (J) length of the myotubes (n = 4). Scale bar = 200 μm (K) Treatment with LY294002 inhibited the PI3K activity (n = 3) (L) The transcriptive levels of myogenic markers in myoblasts after treating with LY294002 (n = 4) (M) The protein levels of myogenic markers in myoblasts after treating with LY294002 (n = 3). For (D) (E) (K), and (M), representative blots are shown and quantitative analyses from three independent experiments are provided in Suppl. Fig. 3. Statistically significant differences are indicated by ∗ where *P*< 0.05 or ∗∗ <0.01 between the indicated groups.

We next evaluated the impact of the PPY@SF/GelMA hydrogel on the myogenic differentiation of C2C12 myoblasts. The RT-qPCR results demonstrated that the treatment with the PPY@SF/GelMA hydrogel significantly increased the expression of myogenic marker genes, including *Mhc*, *Myog*, and *MyoD*. In particular, the mRNA level of *Mhc* in the PPY@SF/GelMA group was significantly 61.2%, 67.4%, 57.5%, and 44.3% higher than that in the PBS, GelMA, SF, and SF/GelMA groups (Fig. 3A). Similarly, the PPY@SF/GelMA hydrogel signficantly up-regulated the expression of *Myog* and *Myod* by 66.7% and 74.7%, respectively, compared with the PBS group (Fig. 3B and C). Western blot analysis confirmed that the treatment with PPY@SF/GelMA hydrogel enhanced the myogenic differentiation of C2C12 cells (Fig. 3D). The protein levels of MHC, MyoG, and MyoD in the PPY@SF/GelMA group were significantly elevated by 103.1%, 198.3%, and 95.2% when contrasted with the PBS group (Suppl. Fig. 3A). To explore the underlying mechanisms, the phosphorylation levels of PI3K were determined (Fig. 3E). There were no statistically significant differences in the PI3K protein levels across the five groups. However, the phospharylation of PI3K in the PPY@SF/GelMA group was elevated by 53.7%, 36.9%, and 10.2% compared to the GelMA group, SF group, and SF/GelMA group, respectively (Suppl. Fig. 3B and C). The results indicated that the treatment with the conductive PPY@SF/GelMA hydrogel activated the PI3K signaling pathway during myoblast differentiation. To further validate the expression of MHC, which is a typical marker of fully differentiated myotubes, immunofluorescence staining was employed. After culturing for 6 d, mature myotubes were observed in all groups; however, the PPY@SF/GelMA group showed more dense staining for MHC, indicating maturation of myotube formation (Fig. 3F). Quantitative analysis revealed that the treatment with PPY@SF/GelMA hydrogel markedly increased the fusion index, numbers, width, and length of myotubes by 44.1%, 52.8%, 44.4%, and 52.6%, respectively, as compared to the PBS group (Fig. 3G–J).

To further elucidate the role of PI3K in myogenic differentiation, myoblasts were treated with the PI3K inhibitor LY294002. A significant reduction in the phosphorylation levels of PI3K was observed, indicating successful inhibition of PI3K activity (Fig. 3K, Suppl. Fig. 4A). RT-qPCR analysis demonstrated that PI3K inhibition abolished the hydrogel-induced up-regulation of myogenic markers. Specifically, the mRNA expression levels of *Mhc*, *Myog*, and *Myod* were significantly decreased by 37.1%, 31.2%, and 52.2%, respectively, in the inhibitor-treated group compared to the vehicle group (Fig. 3L). Consistently, Western blot analysis confirmed that LY294002 treatment significantly down-regulated the protein levels of MHC, MyoG, and MyoD by 29.2%, 63.8%, and 54.2%, respectively, relative to the vehicle group (Fig. 3M, Suppl. Fig. 4B). Therefore, these results suggested that the PPY@SF/GelMA hydrogel effectively enhanced the myogenic differentiation and myotube formation of C2C12 cells.

### 3.4 The PPY@SF/GelMA hydrogel promoted endothelial tube formation of HUVECs

**Fig. 4:**
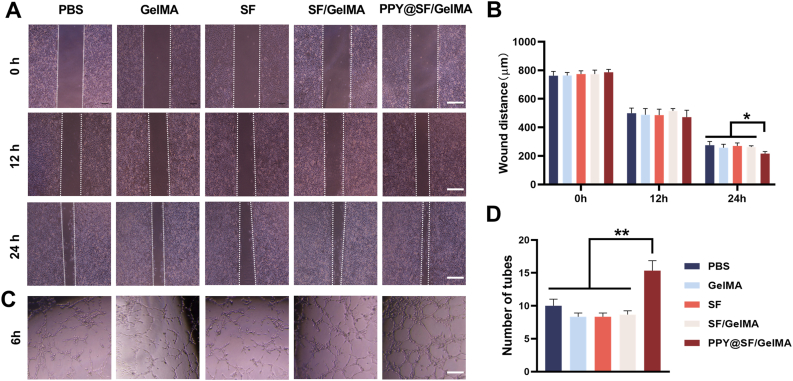
The pro-angiogenic effect of the conductive PPY@SF/GelMA hydrogel on HUVECs (A-B) The cell migration capacity of HUVECs was assessed through scratch assays at 0, 12, and 24 h following exposure to the hydrogel leachate or PBS (n = 3). Scale bar = 200 μm (C-D) The pro-angiogenic effect of the conductive hydrogel was evaluated through endothelial tube formation assays (n = 3). Scale bar = 100 μm. The data are presented as mean ± SD. Statistically significant differences are indicated by ∗ where *P*< 0.05 or ∗∗ <0.01 between the indicated groups.

Angiogenesis plays a critical role in skeletal muscle regeneration, so we investigated the influence of the conductive PPY@SF/GelMA hydrogel on the migration capacity and tube formation of endothelial cells. HUVECs were treated with the leachate from GelMA, SF, SF/GelMA, and PPY@SF/GelMA hydrogels. The wound healing results showed that, after 24 h of the scratch assay, the migration capacity of HUVECs in the PPY@SF/GelMA group was significantly improved, with a respective increase of 21.4%, 16.2%, 20.2%, and 17.8% compared to the PBS, GelMA, SF, and SF/GelMA groups (Fig. 4A and B). The impact of PPY@SF/GelMA hydrogel on angiogenesis was assessed by an endothelial tube formation assay. After culturing with the hydrogel leachated, the number of endothelial tubes in the PPY@SF/GelMA group was significantly 52.3%, 80.3%, 78.1%, and 74.8% higher than that in the PBS, GelMA, SF, and SF/GelMA groups (Fig. 4C and D). These results indicated a pro-angiogenic effect of the conductive PPY@SF/GelMA hydrogel.

### 3.5 The conductive PPY@SF/GelMA hydrogel exhibited excellent *in vivo* biocompatibility

**Fig. 5:**
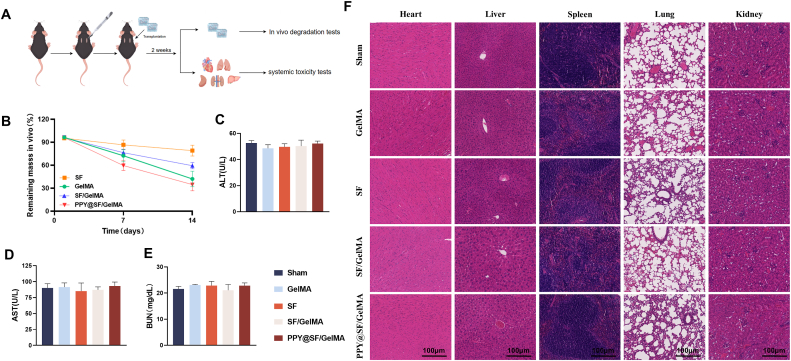
*In vivo* evaluation of degradation profile and biocompatibility of hydrogels (A) Schematic diagram of subcutaneous implantation of GelMA, SF, SF/GelMA, and PPY@SF/GelMA hydrogels (B) *In vivo* degradation profile of the four hydrogels at post-operative days 7 and 14 (n = 4) (C) Quantification of the activity of alanine aminotransferase (ALT) (n = 4) (D) Quantification of the activity of aspartate aminotransferase (AST) (n = 4) (E) Quantification of the concentration of blood urea nitrogen (BUN) (n = 4) (F) Histopathological analysis by H&E staining of major organs (the heart, liver, spleen, lungs, and kidneys) after 14-day subcutaneous implantation (n = 4). Scale bar = 100 μm. The data are presented as mean ± SD.

To evaluate their *in vivo* degradation and biocompatibility, GelMA, SF, SF/GelMA, and PPY@SF/GelMA hydrogels were subcutaneously implanted into the dorsal region of C56BL mice (Fig. 5A). On post-operative days 7 and 14, the hydrogels were harvested and weighed. Consistent with the *in vitro* degradation assay, all four hydrogels exhibited slow degradation rates. Notably, the SF hydrogel displayed the slowest degradation rate, retaining 79.3% of its initial mass by day 14, compared to the GelMA hydrogel at 41.2% and the PPY@SF/GelMA hydrogel at 33.6% (Fig. 5B).

To further assess the *in vivo* biosafety of the conductive hydrogel, the serum levels of ALT, AST, and BUN were quantified (Fig. 5C–E). The results showed no statistically significant differences among the five groups, thereby indicating that the conductive hydrogel had no hepatotoxicity in mice. For the systemic toxicity assessment, major organs, including the heart, liver, spleen, lungs, and kidneys, were harvested for histopathological analysis at 14 days post-implantation. H&E staining revealed no signs of toxicity or adverse effects from the implanted hydrogels compared to the negative control (Fig. 5F). These findings confirm the excellent biocompatibility of the conductive hydrogel, consistent with the *in vitro* cytotoxicity assessments.

### 3.6 Implantation of the conductive PPY@SF/GelMA hydrogel promoted skeletal muscle regeneration and restored motor functions in a mouse VML model

**Fig. 6:**
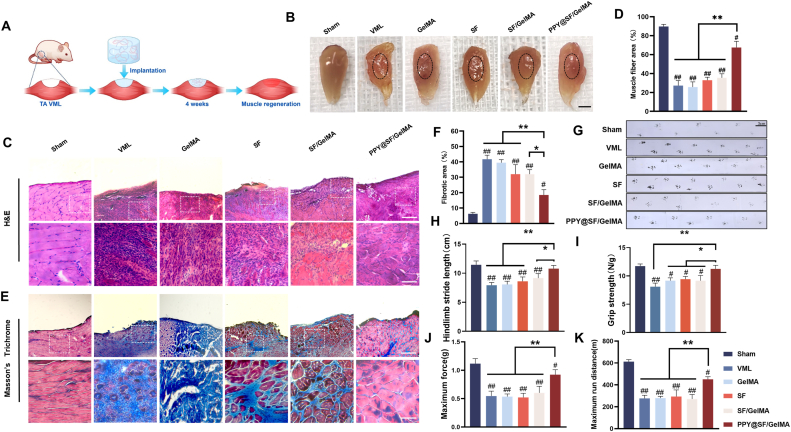
The conductive PPY@SF/GelMA hydrogel restores the functional performance of injured muscle after 4 weeks of VML injury (A) Schematic diagram of skeletal muscle regeneration by the conductive PPY@SF/GelMA hydrogel (B) The gross morphological presentation of the newly regenerated TA muscle post-VML injury (delineated by a black dotted line). Scale bar = 5 mm (C) Representative H&E staining of the newly regenerated myofibers, characterized by the presence of central nuclei. Scale bar = 200 μm (upper panels); Scale bar = 50 μm (lower panels) (D) Quantification analysis of the area occupied by the newly regenerated muscle fibers (n = 4) (E) Representative images of Masson's trichrome staining of collagen deposition. Scale bar = 200 μm (upper panels); Scale bar = 50 μm (lower panels) (F) Quantification of the area of the fibrotic tissue (n = 4) (G&H) The gait analysis of mice at 4-week post-injury (n = 4) (I) The grip strength test of mice at 4-week post-injury (n = 4) (J) Muscle electromyography analysis of mice at 4-week post-injury (n = 4) (K) The running test of mice at 4-week post-injury (n = 4). The data are presented as mean ± SD. Statistically significant differences are indicated by # where *P* < 0.05 or ## where *P*< 0.01 compared to the Sham group; ∗ where *P*< 0.05 or ∗∗ where *P* < 0.01 between the indicated groups.

To assess the regenerative efficacy of the PPY@SF/GelMA hydrogel on skeletal muscle regeneration, a mouse VML model was established by excising approximately 20% volume of the TA muscle and subsequently four groups of hydrogels were implanted at the defect site (Fig. 6A). As shown in the gross morphology images, no signs of infection or severe inflammation were observed in any of the groups after 4 weeks of the surgery. Notably, the PPY@SF/GelMA hydrogel group showed significantly improvement of muscle regeneration at the site of injury (Fig. 6B). H&E staining results revealed a pronounced increase in the density of newly regenerated muscle fibers in the VML defect site in the PPY@SF/GelMA group when compared to the VML and other hydrogel groups (Fig. 6C). Quantitative analysis confirmed that the skeletal muscle fiber area in the PPY@SF/GelMA group was 1.5-fold, 1.6-fold, 1.0-fold, and 92.3% higher than that of the VML, GelMA, SF, and SF/GelMA groups, respectively (Fig. 6D). Furthermore, Masson's trichrome staining was performed to evaluate the presence of fibrosis (indicated by collagen deposition) during the skeletal muscle repair. A strongly positive collagen staining was observed in the defect site of the VML group (Fig. 6E). On the contrary, the PPY@SF/GelMA group showed minimal collagen deposition. Quantitative analysis revealed that the collagen-positive area was 44.3%, 46.8%, 57.7%, and 55.4% lower than that of the VML, GelMA, SF, and SF/GelMA groups, respectively (Fig. 6F).

To evaluate the recovery effect of the skeletal muscle motor functions, a gait analysis, an electromyography analysis, and a running test were performed after 4 weeks of hydrogel implantation. The gait analysis results showed that the mice in the PPY@SF/GelMA group achieved 94.2% of the gait parameters observed in the sham-operated group and a significant improvement of 36.1% compared to the untreated VML group (Fig. 6G and H). Additionally, the results of grip strength tests revealed that the implantation of the PPY@SF/GelMA hydrogel attained 95.9% of the strength recorded in the sham controls, corresponding to a 38.9% increase relative to the VML group (Fig. 6I). The maximum hindlimb force of the PPY@SF/GelMA group was significantly 67.1%, 70.8%, 53.6%, and 75.9% higher than that of the VML, GelMA, SF, and SF/GelMA groups, respectively (Fig. 6J). Meanwhile, the maximum run distance of the PPY@SF/GelMA group was significantly 63.4%, 62.2%, 53.9%, and 65.4% higher than that of the VML, GelMA, SF, and SF/GelMA groups, respectively (Fig. 6K). Though the hindlimb force and run distance of the PPY@SF/GelMA group were lower than those of the Sham group, implantation of the conductive hydrogel effectively restored the motor function of VML-injuried mice. These results indicated that implantation of the conductive PPY@SF/GelMA hydrogel promoted skeletal muscle regeneration while inhibiting tissue fibrosis in the VML defect site.

### 3.7 The conductive PPY@SF/GelMA hydrogel enhanced vascularization in skeletal muscle regeneration

**Fig. 7:**
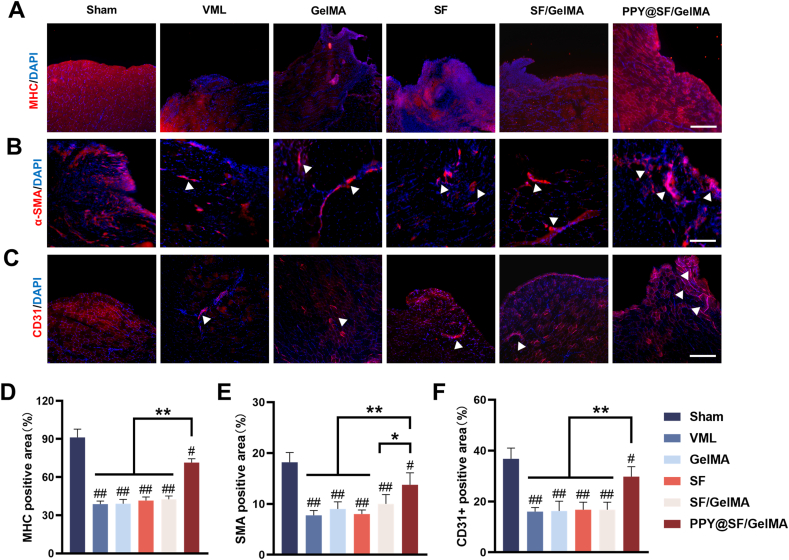
Immunofluorescence staining for *in situ* implantation of PPY@SF/GelMA promoting regeneration of skeletal muscle following VML injury at a 4-week postoperative interval (A) Representative images of immunofluorescence staining for MHC were presented for the VML injury site (B&C) Immunohistochemistry staining of ɑ-SMA and CD31. The arrows denote vessels (D) Quantification of the MHC-positive area (n = 4) (E) Measurement of ɑ-SMA-positive fluorescence area (n = 4) (F) Quantitative assessment of the CD31-positive vessels, n = 6. Scale bar = 200 μm. The data are presented as mean ± SD. Statistically significant differences are indicated by # where *P* < 0.05 or ## where *P*< 0.01 compared to the Sham group; ∗ where *P*< 0.05 or ∗∗ where *P* < 0.01 between the indicated groups.

After 4 weeks of implantation, the PPY@SF/GelMA group exhibited the highest level of MHC-positive myofibers in the defect site, indicating robust muscle regeneration (Fig. 7A). Quantitative analysis revealed that the MHC-positive area in the PPY@SF/GelMA group was increased by 83.2%, 84.1%, 72.1%, and 69.9% relative to the VML, GelMA, SF, and SF/GelMA groups, respectively (Fig. 7D). Furthermore, vascularization is a vital process in the process of skeletal muscle regeneration by maintaining muscle metabolism, facilitating the transport of nutrients, oxygen, and metabolic waste. To assess vascularization in the newly regenerated muscle tissue, we used immunofluorescence staining for CD31 and alpha smooth muscle actin (ɑ**-**SMA) to assess the formation of new blood vessels. In the PPY@SF/GelMA group, a large number of strongly positive CD31^+^ cells were observed in multiple sites, suggesting a robust formation of neovascularization after VML injury (Fig. 7B). Quantitative analysis revealing the percentage of CD31^+^ cells in the PPY@SF/GelMA group was increased 85.9%, 82.6%, 81.2%, and 81.1% compared with that in the VML, GelMA, SF, and SF/GelMA groups, respectively (Fig. 7E). Consistently, immunofluorescence staining for ɑ-SMA demonstrated that implantation of the PPY@SF/GelMA group promoted the formation of ɑ-SMA^+^ blood vessels (Fig. 7C). The number of new blood vessels in the PPY@SF/GelMA group was 85.7%, 52.7%, 71.3%, and 40.5% higher than that in the VML, GelMA, SF, and SF/GelMA groups, respectively (Fig. 7F).

### 3.8 The conductive PPY@SF/GelMA hydrogel suppresses TGF-β1 expression and promoted M2 macrophage polarization

**Fig. 8:**
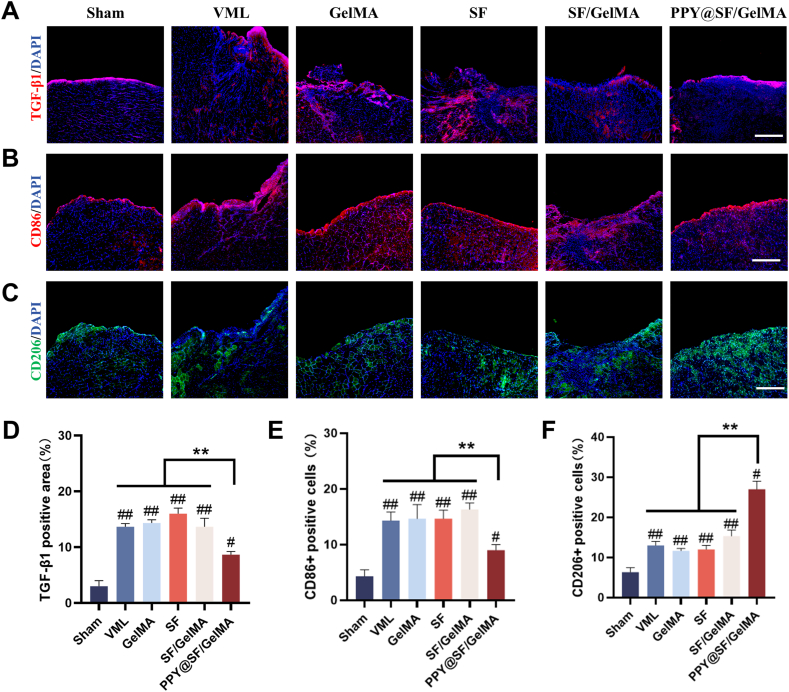
The PPY@SF/GelMA suppressed TGF-β1 expression and promoted M2 macrophage polarization in the regenerated skeletal muscle (A) Representative images of immunofluorescence staining for TGF-β1 in the VML injury site (B-C) Representative images of immunofluorescence staining for CD86 and CD206 (D) Quantification of the TGF-β1 expression (n = 4) (E) Quantitative assessment of CD86^+^ M1 macrophages (n = 4) (F) Quantitative assessment of CD206^+^ M2 macrophages (n = 4). Scale bar = 200 μm. The data are presented as mean ± SD. Statistically significant differences are indicated by # where *P* < 0.05 or ## where *P*< 0.01 compared to the Sham group; ∗ where *P*< 0.05 or ∗∗ where *P* < 0.01 between the indicated groups.

To elucidate the underlying mechanism by which the PPY@SF/GelMA hydrogel inhibits fibrotic tissue formation in regenerated skeletal muscle, immunofluorescence staining for TGF-β1 was conducted (Fig. 8A). The results indicated that the TGF-β1-positive area in the PPY@SF/GelMA group was reduced by 37.8%, 40.2%, 44.5%, and 37.1% compared to the VML, GelMA, SF, and SF/GelMA groups, respectively (Fig. 8D). This reduction in collagen deposition was likely attributed to the decreased expression of TGF-β1. Concurrently, the immunomodulatory effect of the PPY@SF/GelMA hydrogel on M2 macrophage polarization was investigated (Fig. 8B and C). The conductive hydrogel treatment promoted macrophage polarization towards the M2 phenotype, as evidenced by an increase in CD206 expression and a decrease in CD86 expression. Quantitative analysis revealed that the proportion of CD206^+^ cells in the PPY@SF/GelMA group was 101.2%, 84.6%, 108.6%, and 77.9% higher than in the VML, GelMA, SF, and SF/GelMA groups, respectively (Fig. 8E and F).

### 3.9 The conductive PPY@SF/GelMA hydrogel recovered peripheral nerve re-innervation in the newly regenerated skeletal muscle

**Fig. 9:**
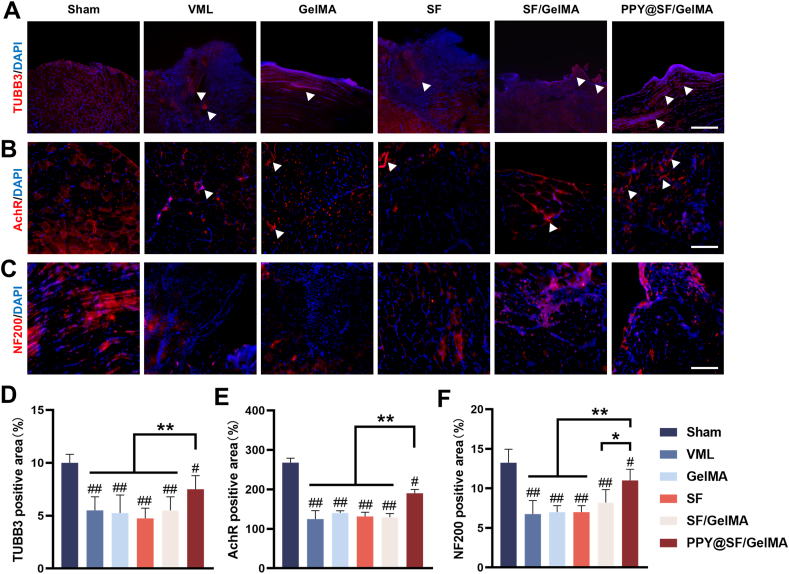
The PPY@SF/GelMA promotes nerve innervation 4 weeks after VML injury (A&B) Immunofluorescence staining of TUBB3 and AchR. The arrows indicate TUBB3 nerves and AchR clusters (C) Immunofluorescence staining of NF200 (D) Quantification of the TUBB3 positive area (n = 4) (E) Measurement of AchR positive fluorescence area (n = 4) (F) quantitative analysis of NF200 fluorescence, n = 6. Scale bar = 200 μm. The data are presented as mean ± SD. Statistically significant differences are indicated by # where *P* < 0.05 or ## where *P*< 0.01 compared to the Sham group; ∗ where *P*< 0.05 or ∗∗ where *P* < 0.01 between the indicated groups.

To further evaluate the effect of the conductive hydrogel on peripheral nerve innervation, samples were stained with antibodies against tubulin beta-III (TUBB3), acetylcholine receptor (AchR) clusters and neurofilament H (NFH). At 4 weeks post-implantation, TUBB3^+^ neurons were observed at the defect site in the PPY@SF/GelMA group (Fig. 9A) and the density showed a 36.3%, 42.7%, 55.6%, and 33.4% increase compared with that in the VML, GelMA, SF, and SF/GelMA groups, respectively (Fig. 9D). Consistently, a few AChR^+^ clusters (Fig. 9B) and robust NFH^+^ nerves (Fig. 9C) were found on the myotubes in the PPY@SF/GelMA group. Quantitatively, the densities of AChR^+^ clusters and NFH^+^ nerves in the PPY@SF/GelMA group were significantly 52.6% (Figs. 9E) and 59.2% (Fig. 9F) higher than that in the VML group. These results indicated that the implantation of PPY@SF/GelMA hydrogel effectively recovered peripheral nerve innervation by improving the formation of neuromuscular junctions.

### 3.10 RNA transcriptome sequencing analysis of newly regenerated TA muscle

**Fig. 10:**
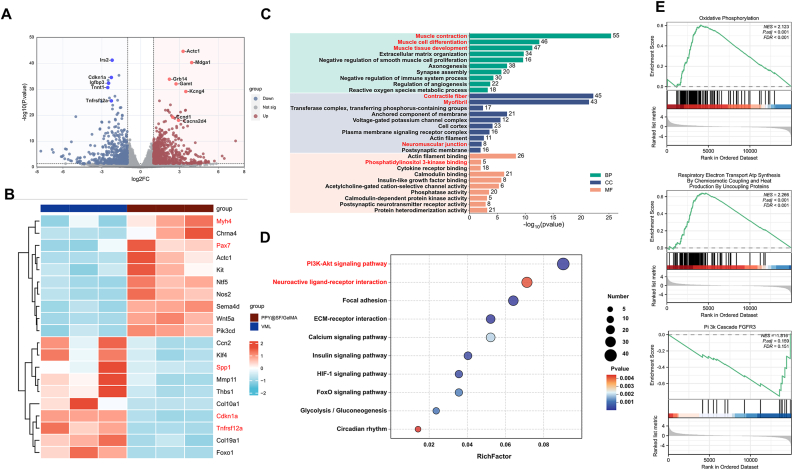
Transcriptomic profiling of the newly regenerated TA muscle in PPY@SF/GelMA hydrogel-treated mice (A) Volcano plots of gene expression levels in the PPY@SF/GelMA versus VML groups (B) Heat maps of differentially expressed genes (DEGs) between the PPY@SF/GelMA and VML groups (C) Gene Ontology (GO) analysis identified biological process (BP), molecular function (MF), and cellular component (CC) from significantly enriched DEGs (D) Top 10 enriched pathways by Kyoto Encyclopedia of Genes and Genomes (KEGG) analysis (E-G) Gene Set Enrichment Analysis (GSEA) analysis of genes associated with oxidative phosphorylation (E), respiratory electron transport ATP synthesis (F), and PI3K cascade FGFR3 (G).

To elucidate the underlying mechanisms by which the PPY@SF/GelMA hydrogel facilitates muscle regeneration and nerve innervation, RNA transcriptome sequencing was performed on newly regenerated TA muscle tissues excised from the injury sites of VML and hydrogel-treated mice. Pairwise comparative volcano plots identified 920 DEGs in the PPY@SF/GelMA group compared to the VML group, with 529 genes being up-regulated and 391 genes being down-regulated (Fig. 10A). The heatmap analysis indicated that PPY@SF/GelMA treatment significantly up-regulated several genes associated with muscle regeneration, such as paired box 7 (*Pax7*) and myosin heavy chain 4 (*Myh4*), while down-regulated genes included secreted phosphoprotein 1 (*Spp1*), cyclin dependent kinase inhibitor 1 A (*Cdkn1a*), and TNF receptor superfamily member 12 A (*Tnfrsf12a*) (Fig. 10B). To gain functional insights into these DEGs, GO enrichment analysis was conducted, encompassing three primary domains: biological process (BP), molecular function (MF), and cellular component (CC). The results demonstrated significant enrichment of DEGs in key biological processes related to muscle differentiation, including muscle cell differentiation, muscle tissue development, and muscle contraction. Moreover, significant terms related to cellular components and molecular functions, such as myofiber, contractile fiber, transferase complex, phosphorus-containing group transfer, and neuromuscular junction, were enriched, suggesting that essential cellular components are associated with muscle function and nerve innervation (Fig. 10C). Notably, KEGG pathway analysis revealed the involvement of the PI3K signaling pathway in the muscle treated with PPY@SF/GelMA (Fig. 10D). Additionally, GSEA analysis confirmed that the PPY@SF/GelMA treatment activated gene sets linked to oxidative phosphorylation, respiratory electron transport ATP synthesis, and the PI3K cascade involving fibroblast growth factor receptor 3 (*Fgfr3*) (Fig. 10E–G).

## 4 Discussion

Skeletal muscle tissue exhibits a diminished regenerative capacity when muscle defect injuries exceed 20% of the total muscle mass. Given that hydrogels can offer an appropriate microenvironment conducive to cell infiltration, growth, and differentiation [26], the application of hydrogel-based muscle tissue engineering is essential for reconstructing the physiological architecture and restoring the normal contractile function of native skeletal muscle. In this study, we developed a dual cross-linked SF/GelMA hydrogel with high conductivity through the incorporation of PPY. The resulting conductive PPY@SF/GelMA hydrogel demonstrated excellent biocompatibility and facilitated the myogenic differentiation of C2C12 cells *in vitro*. Moreover, in a mouse TA VML model the implantation of this conductive hydrogel effectively promoted muscle tissue regeneration, accompanied by active innervation and restoration of normal motor function.

In recent years, a range of conductive biomaterials has been developed for the repair of peripheral nerve tissue. For example, a graphene oxide-containing conductive hydrogel has been reported as a nerve guidance conduit to support the proliferation and differentiation of PC12 neural cells [27]. In this study, a double network hydrogel composed of GelMA and SF was developed to offer appropriate mechanical support and a conducive environment for cellular growth. Through integration with graphene and a gradient of netrin-1, the SF/GelMA hydrogel facilitated the repair of peripheral nerve injuries by promoting axonal extension and remyelination [28]. GelMA, derived from the natural extracellular matrix, is commonly utilized in the fabrication of biocompatible and biodegradable biomaterials. In addition, SF biomaterials demonstrate skeletal muscle-like elasticity, which is essential for myoblast differentiation and myotube maturation [29]. To enhance the regenerative capacity of the SF/GelMA hydrogel for peripheral nerves, we chose to incorporate the conductive material PPY into the SF/GelMA hydrogel, given its prevalent application in the fabrication of artificial nerve conduits [30]. In comparison to GelMA and SF/GelMA hydrogels, the PPY@SF/GelMA hydrogel exhibited superior conductivity while maintaining its inherent porous structure and mechanical properties. The observed enhancement in cross-linking density and mechanical strength can likely be attributed to the incorporation of PPY. The elasticity of the PPY@SF/GelMA hydrogel closely resembles that of natural skeletal muscle tissue. Skeletal muscle tissue, characterized by its high water content, undergoes deformation of approximately 40% during contraction [31]. Previous studies on conductive hydrogel materials for skeletal muscle repair have primarily focused on improving the mechanical properties and electrical conductivity of these hydrogels. Xue et al. reported a conductive GelMA hydrogel scaffold utilizing silver nanowires as the conductive dopant, achieving an electrical conductivity of 2.6 × 10^−3^ S cm^−1^ and a mechanical strength of 10.5 kPa [24]. Notably, although the leachate derived from the PPY@SF/GelMA hydrogel was non-conductive, it still significantly enhanced myogenic differentiation and angiogenic activity *in vitro*. This indicates that the superior biological performance was mediated by soluble physicochemical signals rather than direct electrical stimulation. Trace dopant ions and low-molecular-weight PPY oligomers released during extraction may act as chemical signaling molecules, while micro- or nanoscale PPY fragments can interact with the cell membrane, altering membrane curvature and triggering integrin-mediated mechanotransduction that drives PI3K phosphorylation [32]. This mechanism is consistent with the increased PI3K activation and the PI3K pathway enrichment, suggesting that material-derived surface effects can regulate cell fate independently of bulk conductivity.

Given the critical role of myoblasts in myotube maturation and skeletal muscle regeneration, we thoroughly investigated the effects of the conductive PPY@SF/GelMA hydrogel on myoblast differentiation. *In vitro* treatment of C2C12 cells with leachate from the PPY@SF/GelMA hydrogel resulted in a significant upregulation of myogenic markers at both the gene and protein levels. Furthermore, implantation of the PPY@SF/GelMA hydrogel at the defect site in C57BL/6 mice markedly enhanced muscle tissue repair, as demonstrated by pronounced immunofluorescence staining for MHC. In addition to the beneficial impact on neural differentiation, PPY has been documented to enhance the adhesion of C2C12 myoblasts to polymer scaffolds and to promote myoblast differentiation by up-regulating myogenic gene expression and facilitating myotube fusion [33]. Zhou et al. reported a muscle-adhesive conductive hydrogel incorporating polypyrrole/polydopamine nanoparticles that effectively accelerated skeletal muscle repair within a full-thickness defect model [34]. In this study, Masson's trichrome staining demonstrated that, in comparison to the untreated and GelMA groups, the regenerated tissue in the PPY@SF/GelMA group consisted of skeletal muscle fibers rather than fibrotic scar tissue, which is crucial for the restoration of muscle motor function. The underlying mechanism may involve the inhibition of the TGF-β1 signaling pathway, as indicated by the reduced expression of TGF-β1 in the regenerated muscle tissue [35]. Additionally, the presence of the SF component within the conductive hydrogel may contribute to decreased collagen deposition, as SF-based scaffolds have been shown to effectively suppress scar formation in a rabbit ear hypertrophic scar model [36]. Consequently, both SF and PPy within the double network conductive hydrogel facilitated enhanced myoblast differentiation *in vitro* and promoted myotube formation *in vivo*. This study was limited to a single observation time point at 4 weeks. To validate the translational potential of the conductive hydrogel for reconstructing larger muscle masses, large animal models comparable to humans, such as porcine or ovine models, will be utilized to establish a VML damage model via surgical ablation. The regenerative process and fibrotic tissue formation will be monitored for up to 180 days to provide more reliable data for human translation [37]. Therefore, data from earlier phases (e.g., 1–2 weeks, capturing acute inflammation and early axonal sprouting) and later phases (e.g., 8–12 weeks, assessing long-term myofiber maturation and functional stability) will provide a more complete temporal understanding of the repair dynamics.

Furthermore, the animal experiments indicated that treatment with the conductive PPY@SF/GelMA hydrogel significantly enhanced skeletal muscle angiogenesis, as well as the superior muscle contraction strength. At four weeks post-surgery, the conductive hydrogel promoted vascularization in the regenerated muscle tissue, as evidenced by immunofluorescence staining for CD31, a well-established marker for new capillary vessels. Consistent with our findings, Fan et al. demonstrated that a PPY-based conductive interpenetrating network hydrogel facilitated local vascular and nerve regeneration in a rat model of diabetic wound healing through the activation of intracellular calcium-related signaling pathways [38]. In addition, co-culture of endothelial cells and myoblasts on a pre-vascularized electrospun scaffold resulted in an increase in striated MHC-positive myotubes and an upregulation of myogenic-specific genes in myoblasts, suggesting that endothelial cell-mediated vascularization positively influences myoblast differentiation and myotube formation [39]. Moreover, during skeletal muscle regeneration, an increased number of blood vessels is essential for enhancing the quality of regenerated skeletal muscle tissue by ensuring adequate oxygen supply and efficient metabolite removal [40]. In ischemic limbs, the restoration of endothelial cellular functions through the delivery of vascular endothelial growth factor and delta-like 4 has been demonstrated to effectively promote the formation of capillaries and arterioles, thereby accelerating vascularization and muscle regeneration [41].

Neuromuscular junctions are crucial for the regulation of skeletal muscle functions, encompassing processes such as myogenesis, maturation, and regeneration. Recent research has indicated that VML injury not only leads to the loss of myofibers but also causes secondary denervation of neuromuscular junctions, thereby resulting in chronic functional impairment and suboptimal recovery of skeletal muscle [42]. In this study, we examined the impact of the PPY@SF/GelMA conductive hydrogel on the formation of neuromuscular junctions at the VML injury site, as demonstrated by the presence of newly formed AChR^+^ clusters and NFH^+^ nerves. RNA-seq analysis revealed that the PPY@SF/GelMA hydrogel may activate the PI3K signaling pathway in newly regenerated muscle tissues, thereby possibly enhancing the thickness of the myelin sheath surrounding axons and improving the motor function of skeletal muscle [32]. The regenerative effects of the conductive hydrogel on neuronal myelination may be mediated through targeting the interleukin 17 (IL-17) receptor A and subsequently activating the IL-17 signaling pathway [19]. Furthermore, appropriate electrical stimulation can facilitate the repair of innervated skeletal muscle by reorganizing the myoblast cytoskeleton to align with the direction of the electric field. The combination of a PPY-integrated conductive scaffold and electrical stimulation effectively promotes axonal regeneration and remyelination, suggesting significant potential for treating neural injuries [43]. Myoblasts and neural cells spontaneously generate rhythmic electrical activities during differentiation. These intrinsic bioelectrical cues are crucial for myotube fusion and neuromuscular synapse formation [44]. Non-conductive scaffolds such as pure GelMA act as insulators that disrupt intercellular electrical communication. In contrast, the PPY@SF/GelMA conductive hydrogel serves as a bioelectrical bridge through its conductive polymer network, reducing intercellular resistance and facilitating the propagation of action potentials and local electric fields [45]. Future research will aim to elucidate the response of neuronal cells, such as Schwann cells and PC12 cells, to the conductivity of the hydrogel and explore the practical applications of conductive biomaterials combined with electrical stimulation in peripheral nerve regeneration.

Consistent with our findings, previous studies have documented the pro-regenerative impact of motor neurons on skeletal muscle regeneration following VML. The implantation of a pre-innervated tissue construct into a rat VML defect significantly improved the formation of neuromuscular junctions and the vascularization of the regenerated muscle tissue [46]. Co-culture of motor neurons and skeletal myocytes not only enhanced the expression of genes associated with neuromuscular junctions but also facilitated myofiber fusion and elongation, suggesting that motor neurons create a conducive molecular environment to support the maturation of skeletal muscle [47]. PPY-mediated neuromuscular electrical stimulation can effectively enhance satellite stem cell proliferation and facilitate their fusion with mature myofibers by reducing superoxide anion production, thereby enhancing the regenerative capacity of skeletal muscle at the injury site [48]. Additionally, to evaluate their role in the restoration of muscle function following VML injury, neural cells were loaded into a three-dimensional bioprinted skeletal muscle construct. Upon implantation into muscle defects, it was observed a rapid formation of neuromuscular junctions and functional innervation within the newly organized muscle tissue, leading to an effective recovery of normal muscle weight and function [49]. However, the underlying mechanisms by which re-innervation of neuromuscular junctions contributes to skeletal muscle regeneration remain unclear. The prostaglandin-degrading enzyme, 15-hydroxyprostaglandin dehydrogenase (15-PGDH), has been reported as a promising target for the restoration of neuromuscular connectivity following both acute and chronic denervation, because administration of a small-molecule inhibitor of 15-PGDH in aged denervated mice significantly enhanced motor axon regeneration, resulting in a substantial improvement in the strength of denervated muscles [50]. Consequently, our future studies will aim to elucidate the role of neuromuscular junctions in skeletal muscle regeneration following VML injury.

Despite the robust regenerative capacity demonstrated by the PPY@SF/GelMA scaffold, the present investigation remains constrained by specific methodological limitations that must be acknowledged. Recent evidence indicates that skeletal muscle cells can direct spinal cord neural stem cells toward a neuronal fate while suppressing glial differentiation, leading to enhanced synaptogenesis and axonal outgrowth [51]. However, the current experimental design lacks direct *in vitro* neuro-muscle cell co-culture models, which are essential to mechanistically map the synaptic cross-talk between sprouting axons and nascent myotubes. Meanwhile, our SF/GelMA double-network hydrogel provides a biomimetic and biocompatible foundation for tissue regeneration yet fails to guide motor axon growth due to insufficient structural orientation [52]. Future research will aim to develop hierarchically oriented biomaterials that exhibit both superior electrical conductivity and mechanical stability. This will be achieved through the application of electrospinning [53] and 3D printing technologies [54], with the aim of enhancing nerve regeneration and facilitating the recovery of muscle function. Furthermore, the current design did not incorporate external electrical stimulation that may further enhance the efficiency of neuromuscular regeneration. Lyu et al. demonstrated that an ultrasound-activated piezoelectric hydrogel was able to promote functional muscle repair by orchestrating myogenesis and reinnervation without implanted electrodes, highlighting the translational potential of non-invasive electroactive strategies [55]. Furthermore, While the distinct spatial colocalization of AChR clusters with TUBB3/NFH-positive neural axons provides compelling evidence of structural re-innervation at the defect site, the absence of direct *in vivo* functional assessments, such as continuous electromyography (EMG) or voltage-clamp fluorometry (VCF), limits the depth of our mechanistic conclusions regarding signal transduction. Consequently, future investigations will focus on the mechanistic interactions governing the interplay between regenerating muscle fibers and peripheral nerves, integrating co-culture systems and advanced electrophysiological monitoring (e.g., EMG and VCF) to definitively validate the functional restoration of the neuromotor axis [56].

## 5 Conclusion

In this study, we developed an innovative conductive double network hydrogel, PPY@SF/GelMA, which exhibits promising potential as a regenerative biomaterial for the treatment of severe skeletal muscle injuries. Compared to pure GelMA and SF hydrogels, the dual network structure of the PPY@SF/GelMA hydrogel enhances its mechanical support, swelling properties, and conductive performance. *In vitro* experiments revealed that the PPY@SF/GelMA hydrogel significantly facilitates the myogenic differentiation of C2C12 cells. Treatment with the conductive hydrogel enhanced endothelial cell migration and angiogenic differentiation. In an *in vivo* mouse VML model, the implantation of PPY@SF/GelMA hydrogel facilitated muscle fiber maturation, reduced matrix collagen deposition, and accelerated neovascularization. Notably, treatment with PPY@SF/GelMA hydrogel significantly promoted the regeneration of neuromuscular junctions at the defect site, thereby restoring myoelectric activity and motor function in mice with VML injuries. Consequently, the conductive double-crosslinking PPY@SF/GelMA hydrogel represents an effective therapeutic approach for repairing skeletal muscle with accelerated peripheral nerve re-innervation following extensive injuries.

## CRediT authorship contribution statement

Dimulati Maimaiti: Data curation, Formal analysis, Writing - original draft. Ziying He: Data curation, Formal analysis, Writing - review & editing. Jinuo Liu: Data curation, Formal analysis, Writing - review & editing. Shihan Gao: Data curation, Writing - review & editing. Guanyu Yang: Data curation, Writing - review & editing. Ce Qi: Data curation, Writing - review & editing. Kai Meng: Data curation, Writing - review & editing. Fan He: Formal analysis, Funding acquisition, Investigation, Writing - original draft, review & editing. Huijing Zhao: Formal analysis, Investigation, Writing - original draft, review & editing. Xi Chen: Conceptualization, Project administration, Supervision, Formal analysis, Funding acquisition, Writing - original draft, review & editing.

## Ethics

All animal experiments were approved by the Ethics Committee of Soochow University (SUDA20240927A05), and all operations were performed according to the National Institutes of Health guidelines.

## Declaration of generative AI in scientific writing statement

No generative artificial intelligence (AI) or AI-assisted technologies were used in the preparation of this manuscript.

## Funding statement

This study was supported by the 10.13039/501100001809National Natural Science Foundation of China (82072410); the 10.13039/501100004608Natural Science Foundation of Jiangsu Province (BK20220046); Suzhou Applied Basic Research Programs (Health Care) of Science and Technology Innovation Projects (SYW2024069); Top Talent of Changzhou “The 14th Five-Year Plan” High-Level Health Talents Training Project (2024CZBJ011); Science program of Jiangsu Province Administration for Market Regulation (KJ2024010); Jiangsu Province “333Project” Talent Project; Key Laboratory of Orthopaedics of Suzhou (SZS2022017); Priority Academic Program Development of Jiangsu Higher Education Institutions (PAPD).

## Declaration of competing interest

The authors declare that they have no known competing financial interests or personal relationships that could have appeared to influence the work reported in this paper.

## Data Availability

The data that supports the findings of this study are available in the supplementary materials. Data are available from the corresponding author upon reasonable request.
