# Supplementary material for: Re-innervation of neuromuscular junctions by a conductive polypyrrole/silk fibroin/GelMA hydrogel facilitated functional skeletal muscle regeneration following volumetric muscle loss
Source: J Orthop Translat. 2026 May 15;58:101128. doi: 10.1016/j.jot.2026.101128 (PMC13199897; doi:10.1016/j.jot.2026.101128)
Supplement: Multimedia component 1 [file mmc1.docx]

**Supplementary Figure 1**


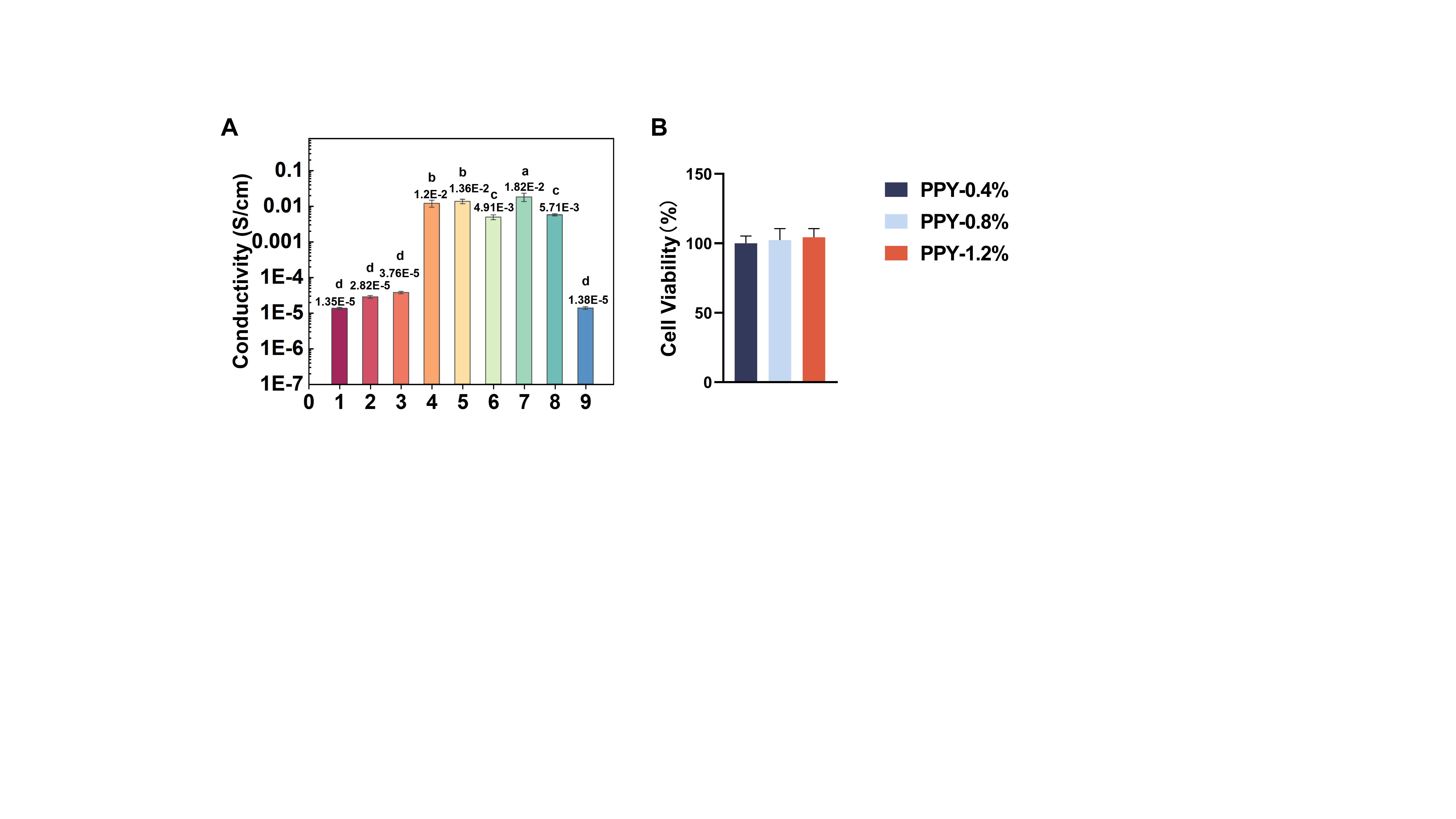


**Suppl. Fig. 1**. Optimizing the preparation of the PPY@SF/GelMA hydrogel containing different concentrations of PPY. Three conductive hydrogels were prepared in preliminary experiments by incubating them in pyrrole solutions of 0.4%, 0.8%, and 1.2% for 30, 60, and 90 min, respectively. (A) The conductivity of three hydrogels. (B) Cell viability of C2C12 myoblasts was quantified using the CCK-8 assay (n=4).

**Supplementary Figure 2**

**
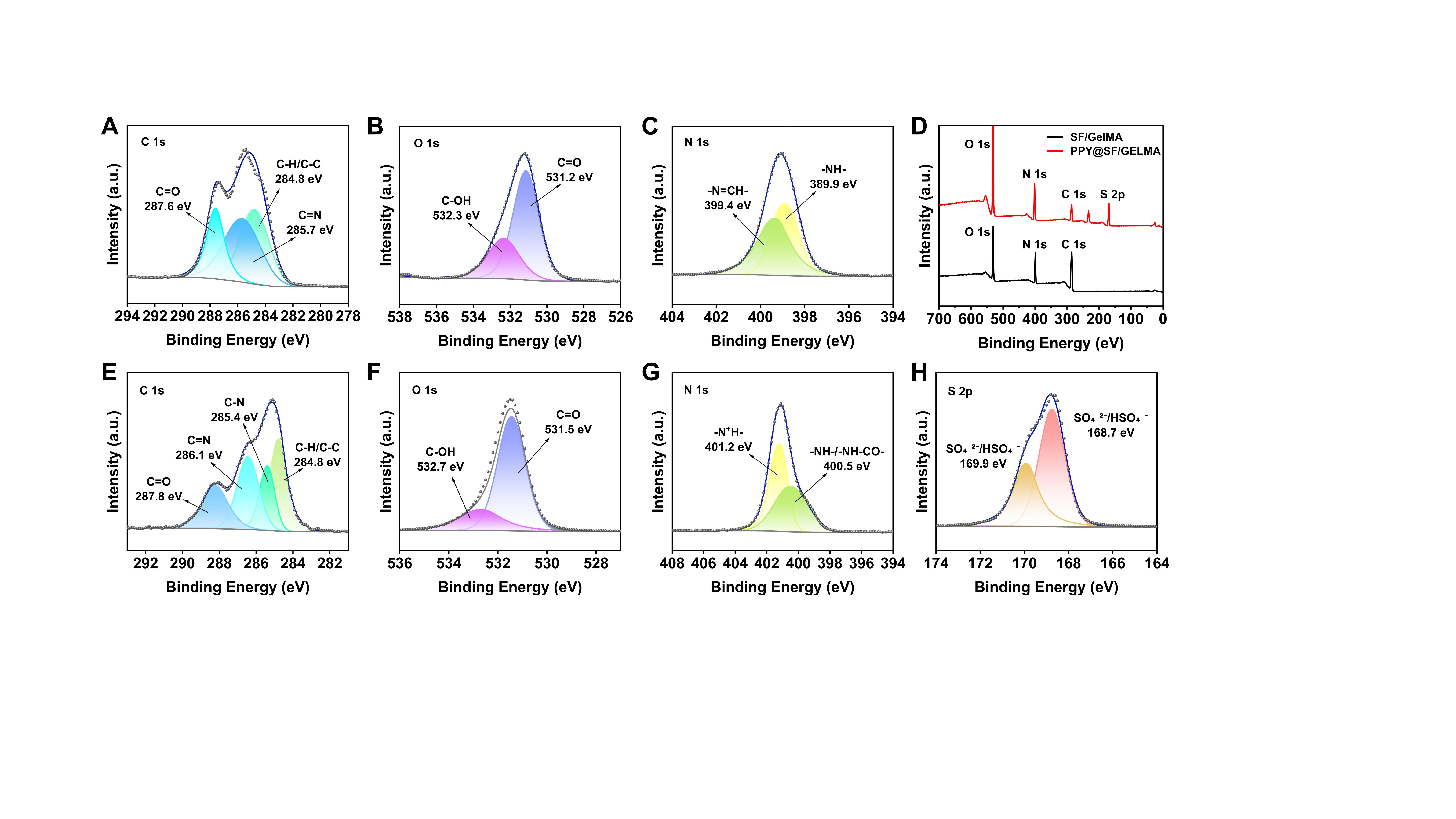
**

**Suppl. Fig. 2.** XPS spectra of the SF/GelMA and PPY@SF/GelMA hydrogels. (A-C) XPS spectra of the SF/GelMA hydrogel: (A) C 1s, (B) O 1s, and (C) N 1s. (D) Survey spectra comparison of the SF/GelMA and PPY@SF/GelMA hydrogels. XPS spectra of the PPy@SF/GelMA hydrogel: (E) C 1s, (F) O 1s, (G) N 1s, and (H) S 2p.

**Supplementary Figure 3**

**
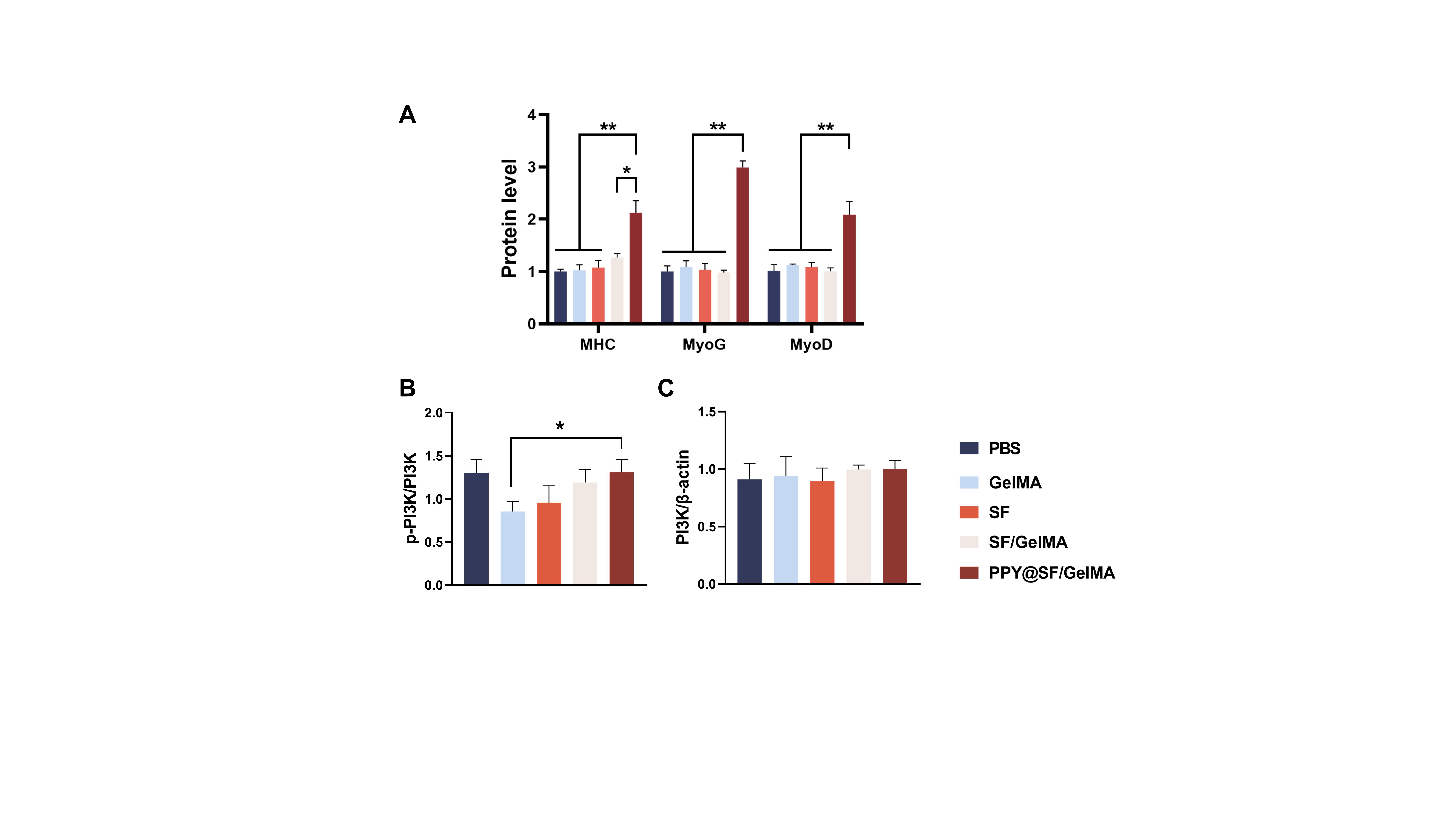
**

**Suppl. Fig. 3.** Quantification of protein expression of myogenic differentiation factors and PI3K. (A) The protein levels of MHC, MyoG and MyoD were examined by Western blot (n = 3). (B) The phosphorylation levels of PI3K were normalized to total protein PI3K (n = 3). (C) The protein levels of PI3K were normalized to β-actin (n = 3). Statistically significant differences are indicated by * where *P* < 0.05 or ** < 0.01 between the indicated groups.

**Supplementary Figure 4**

**
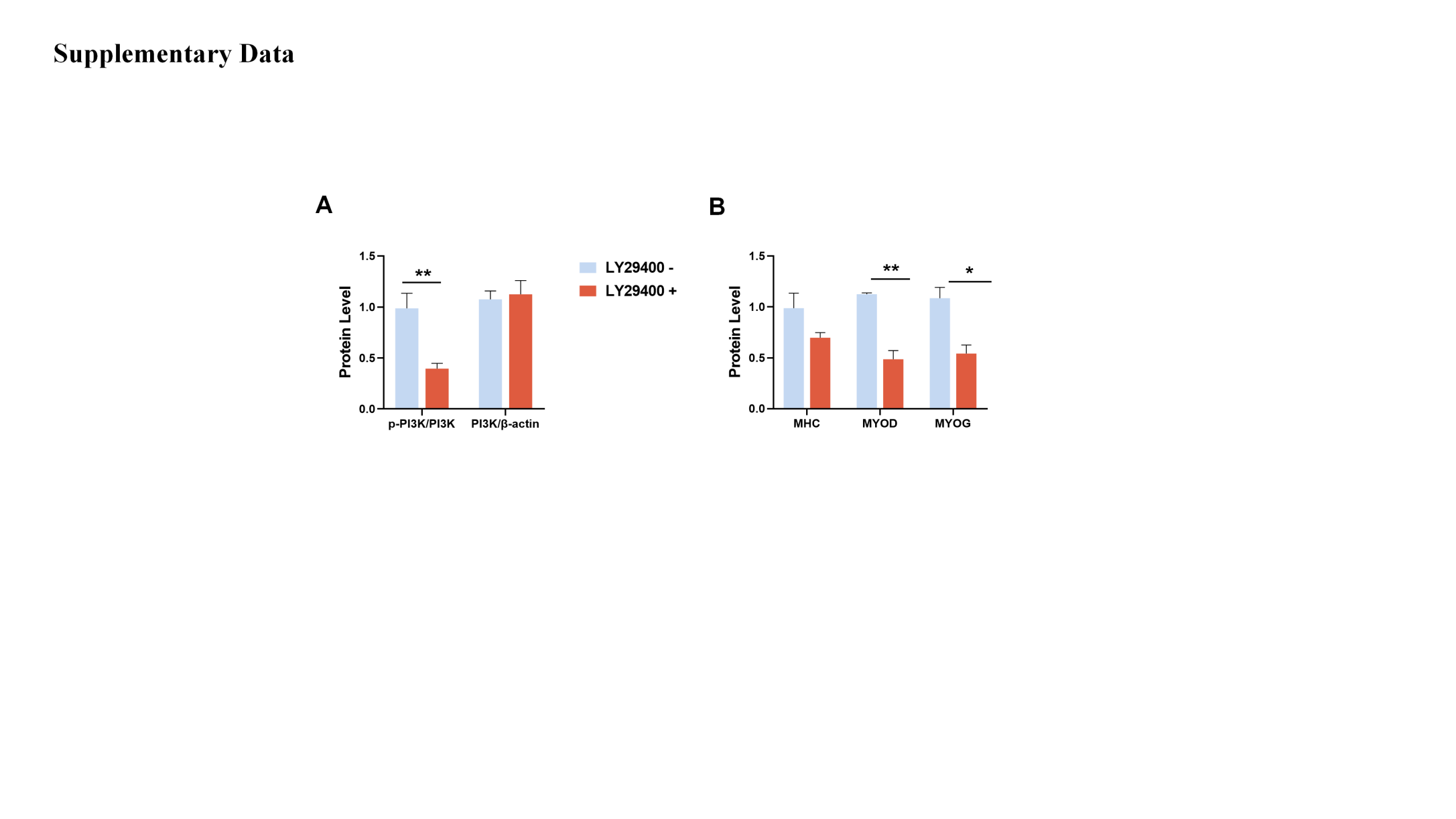
**

**Suppl. Fig. 4.** Treatment with LY294002 inhibited the myogenic differentiation of myoblasts. (A) The phosphorylation and protein levels of PI3K in myoblasts after treating with LY294002 (n = 3). (B) The protein levels of MHC, MyoG and MyoD in myoblasts after treating with LY294002 (n = 3). Statistically significant differences are indicated by * where *P* < 0.05 or ** < 0.01 between the indicated groups.

**Supplementary Table 1.** Primers used for quantitative real-time qRT-PCR.

| Gene | Forward primer sequence (5’ -3’) | Reverse primer sequence (5’-3’) |
| --- | --- | --- |
| *Gapdh* | CCTCGTCCCGTAGACAAAATG | TGAGGTCAATGAAGGGGTCGT |
| *Mhc* | GGAGAAGAAGCAGCGGAAATT | CAGGTCACTGGCTGCCTGTTC |
| *MyoG* | CTTGCTCAGCTCCCTCAACCA | CAGACATATCCTCCACCGTGA |
| *MyoD* | GTGGCGACTCAGATGCATCCA | AGATGCGCTCCACTATGCTG |

**Supplementary Table 2.** Antibodies used for Immunofluorescence staining.

| Antibody | Vendor | Catalog Number | Dilution |
| --- | --- | --- | --- |
| MHC | Abways | CY6635 | 1:200 |
| TUBB3 | Abways | AB0043 | 1:200 |
| α-SMA | Abways | CY5295 | 1:200 |
| AchR | Immunoway | YT5555 | 1:200 |
| NF200 | ABclonal | A19084 | 1:200 |
| CD31 | ABclonal | YM8079 | 1:200 |
| TGF-β1 | Abcam | ab66043 | 1:200 |
| CD206 | ABclonal | A8301 | 1:200 |
| CD86 | ABclonal | A16805 | 1:200 |
| goat anti-mouse IgG (H&L) | Abcam | ab150115 | 1:200 |

**Supplementary Table 3.** Antibodies used for Western blot.

| Antibody | Vendor | Catalog Number | Dilution |
| --- | --- | --- | --- |
| MHC | Abways | CY6635 | 1:2000 |
| MyoG | Abways | CY8830 | 1:2000 |
| MyoD | Immunoway | YT5336 | 1:2000 |
| PI3K | HUABIO | SU04-07 | 1:2000 |
| p-PI3K | HUABIO | PSH01-38 | 1:2000 |
| β-actin | Abcam | ab8227 | 1:300 |
| goat anti-rabbit IgG (H&L) | Abcam | ab150079 | 1:2000 |

**Supplementary Table 4.** Preparation parameters of PPY@SF/GelMA hydrogel.

| Pyrrole solution concentration（%） | Immersing time in pyrrole solution （min） | Immersing time in ammonium persulfate solution （min） |
| --- | --- | --- |
| 0.4 | 30 | 30 |
| 0.4 | 45 | 60 |
| 0.4 | 60 | 90 |
| 0.8 | 30 | 60 |
| 0.8 | 45 | 90 |
| 0.8 | 60 | 30 |
| 1.2 | 30 | 90 |
| 1.2 | 45 | 30 |
| 1.2 | 60 | 60 |

**Supplementary Table 5.** Compression properties of four hydrogels.

| Sample | Maximum compression strength（kPa） | Maximum compressive strain（%） | Modulus of compression（kPa） |
| --- | --- | --- | --- |
| SF | 122.61 ± 19.22 | 22.03 ± 1.92 | 1.34 ± 0.02 |
| GelMA | 55.99 ± 12.37 | 56.00 ± 4.00 | 0.11 ± 0.01 |
| SF/GelMA | 187.42 ± 16.59 | 41.80 ± 2.03 | 2.02 ± 0.04 |
| PPY@SF/GelMA | 212.47 ± 8.60 | 49.75 ± 0.62 | 3.22 ± 0.04 |
